# Comparison of SSR and SNP Markers in Estimation of Genetic Diversity and Population Structure of Indian Rice Varieties

**DOI:** 10.1371/journal.pone.0084136

**Published:** 2013-12-19

**Authors:** Nivedita Singh, Debjani Roy Choudhury, Amit Kumar Singh, Sundeep Kumar, Kalyani Srinivasan, R. K. Tyagi, N. K. Singh, Rakesh Singh

**Affiliations:** 1 Division of Genomic Resources, National Bureau of Plant Genetic Resources, New Delhi, Delhi, India; 2 Germplasm Conservation Division, National Bureau of Plant Genetic Resources, New Delhi, Delhi, India; 3 National Research Centre on Plant Biotechnology, Indian Agricultural Research Institute, New Delhi, Delhi, India; Georgia Institute of Technology, United States of America

## Abstract

Simple sequence repeat (SSR) and Single Nucleotide Polymorphic (SNP), the two most robust markers for identifying rice varieties were compared for assessment of genetic diversity and population structure. Total 375 varieties of rice from various regions of India archived at the Indian National GeneBank, NBPGR, New Delhi, were analyzed using thirty six genetic markers, each of hypervariable SSR (HvSSR) and SNP which were distributed across 12 rice chromosomes. A total of 80 alleles were amplified with the SSR markers with an average of 2.22 alleles per locus whereas, 72 alleles were amplified with SNP markers. Polymorphic information content (PIC) values for HvSSR ranged from 0.04 to 0.5 with an average of 0.25. In the case of SNP markers, PIC values ranged from 0.03 to 0.37 with an average of 0.23. Genetic relatedness among the varieties was studied; utilizing an unrooted tree all the genotypes were grouped into three major clusters with both SSR and SNP markers. Analysis of molecular variance (AMOVA) indicated that maximum diversity was partitioned between and within individual level but not between populations. Principal coordinate analysis (PCoA) with SSR markers showed that genotypes were uniformly distributed across the two axes with 13.33% of cumulative variation whereas, in case of SNP markers varieties were grouped into three broad groups across two axes with 45.20% of cumulative variation. Population structure were tested using K values from 1 to 20, but there was no clear population structure, therefore Ln(PD) derived Δk was plotted against the K to determine the number of populations. In case of SSR maximum Δk was at K=5 whereas, in case of SNP maximum Δk was found at K=15, suggesting that resolution of population was higher with SNP markers, but SSR were more efficient for diversity analysis.

## Introduction

 Rice (*Oryza sativa* L.) is a staple food crop in the world and accounts for 21, 14 and 2% of global energy, protein and fat supply, respectively [1]. It serves as a model plant for genetic breeding and genomics research. Rice is rich in genetic diversity at both interspecific and intraspecific levels. Three subspecies; *indica*, *japonica* and *javanica* constitute a large reservoir of rice germplasm including a variety of local landraces and cultivars [2,3]. Knowledge regarding the extent of genetic variation and genetic relationships between genotypes are important considerations for designing effective breeding and conservation programmes. 

Molecular markers allow precise and rapid varietal identification, which has been proved to be an efficient tool for crop germplasm characterization, collection and management. Earlier RAPD, ISSR and AFLP have been used very frequently for fingerprinting and characterization of varieties and germplasm accessions of different crop species. Since these markers can be utilized without prior genomic information on the target crop for analysis, they were generally used as markers of choice. But after year 2000 the locus specific markers such as Simple Sequence Repeat (SSR) got its preferential application in cultivar identification in many crops, such as grape [4], potato [5], rape [6], rice [7], almond [8], apple [9] and wheat [10]. With the sequencing of several genomes and the possibility of revealing single nucleotide polymorphism (SNP) markers *en masse*, SNPs are gaining importance in diversity studies [11,12]. The primary advantages of these markers are that they occur in genomes at a much higher frequency than SSRs, with close to one SNP being observed per 140 bp in rice [13], and that they can be genotyped in high throughput systems with a high multiplex ratio. The polymorphisms of SSR and SNP are generated via different mechanisms (replication slippage for SSRs vs. point mutation for SNPs) and the two marker types can therefore provide different views of the structure of a given population. Single nucleotide polymorphisms are valuable markers for the construction of high-resolution genetic maps, for the study of population structure, and for the discovery of marker–trait relationships in association-mapping experiments. Application of SNP markers on plant cultivar identification have been reported, in grape [14], grapevine [15], melon [16] and rice [17].

The present study was conducted to compare the two most important and preferred genetic markers, SSRs and SNPs for assessment of genetic variability and population structure among 375 rice varieties tested under distinct, uniform and stable (DUS-tested) system/ released and notified varieties (RV) by Indian system.

## Materials and Methods

### Plant Materials

Seed samples of 231 DUS-tested, 130 DUS-tested/released varieties and 14 released varieties of rice from different parts of India were received from Indian National Genebank, National Bureau of Plant Genetic Resources (NBPGR), New Delhi. The details of each variety along with passport data (national ID i.e. Indigenous Collection (IC) number, State, local name, pedigree, regions), status (DUS/RV) and important traits are given in Table S1. 

### DNA Extraction from Rice Seed

Six (6) to nine (9) seeds of each variety were dehusked and used for DNA isolation using QIAGEN DNeasy plant mini kit (Hilden, Germany). Kernels were ground into fine powder using tissue lyser (Tissue lyser II Retsch, Germany) with a tissue lyser adapter set (QIAGENq). DNA extraction procedure was as per manufacturer’s protocol.

### Genotyping of Rice Varieties using SSR Markers

One hundred and twenty highly variable SSR (HvSSR) marker loci with repeat lengths of 51-70bp located across all twelve chromosomes of rice [18] were selected for initial screening. Ten markers located on long and short arm of each rice chromosome were selected so that total genome can be covered effectively. 

Gradient PCR was set for each primer with selected rice samples to standardize the temperature of amplification (Ta). Out of 120 HvSSR primers, 36 primers which were showing good amplification were selected for final study. 

Genomic DNA of all the 375 varieties was diluted to prepare working stocks of 10 ng/µl. PCR reaction was set in a total volume of 10µl containing 2µl genomic DNA (10ng/µl), 1µl of 10X buffer, 0.8 µl of 25 mM MgCl_2_, 0.2µl of 10mM dNTPs, 0.2 µl of each primer (10nmol), 0.2 µl of Taq DNA polymerase (Fermentas, Life Sciences, USA) and 5.6 µl distilled water. Amplification was performed in a thermocycler using following program; Initial denaturation at 94°C for 4 min followed by 36 cycles of 94°C for 30s, Ta for 45s, 72°C for 1 min and final extension at 72°C for 10 min. The amplified products were analyzed on 4% Metaphor agarose gel for 4 hrs at constant supply of 120V. Gel pictures were recorded using Gel Documentation System (Alpha Imager®, USA).

### Genotyping of Rice Varieties using SNP Markers

The Sequenom Mass ARRAY system (Sequenom Inc., San Diego, CA, USA) uses matrix assisted laser desorption ionization-time of flight (MALDI-TOF) mass spectrometer for accurate detection of SNPs in a high-throughput manner (www. sequenom.com). Sequenom Mass ARRAY multiplex assays were designed for 36 SNPs (iPLEX gold chemistry), representing conserved single-copy rice genes [19], taking three genes per rice chromosome. The 36-plex assays were designed and validated by Sequenom Corporation (San Diego). The 30-mer pre-amplification primers and variable length genotyping primers generated by the Assay Design 3.1 software were procured and used for the validation of SNPs according to the Sequenom user manual. 

### Statistical Analyses

The SSR profiles were scored based on the size (bp) of fragments amplified across all the 375 varieties. The weak gel bands of negligible intensity and smeared bands were excluded from the final data analysis. In case of SNP, the Mass ARRAY Typer 3.4 Software was used for the visualization of SNPs and allele calling. The major allele frequency, gene diversity, heterozygosity and PIC for each locus were calculated for both SSR and SNP markers using Power Marker 3.5 [20]. In addition, genetic distances [21] across the genotypes and neighbor-joining (NJ) tree were calculated using Power Marker 3.5 [20]. The dissimilarity matrix generated by Power Marker was used to construct un-weighted neighbour joining tree using DARwin software 5.0.158 [22]. 

Principle Coordinate Analysis (PCoA) and Analysis of Molecular Variance (AMOVA) were performed using software GenAlEx V6.5 [23]. In case of SNP data, the bases were numerically coded as follows: A=1, C=2, G=3, T=4 and missing data was coded as 0 as suggested in GenAlEx V6.5 user manual [23]. Software STRUCTURE V2.3.1 was applied to infer historical lineages that show clusters of similar genotypes [24]. The membership of each genotype was run for range of genetic clusters from value of K= 1 to 20 with the admixture model and correlated allele frequency. For each K it was replicated 3 times. Each run was implemented with a burn-in period of 100,000 steps followed by 100,000 Monte Carlo Markov Chain replicates [24]. Ln(PD) derived for each K and then plotted to find the plateau of the ΔK values [25]. Online available programme “structure harvester” was used (http://taylor0. biology.ucla.edu) to calculate final population structure. The proportion of the genome of an individual that belongs to each inferred population (admixture) was also estimated.

## Results

The present study was conducted on 375 *indica* rice varieties which included DUS tested as well as released and notified varieties from eighteen major rice growing states of India and varieties released and notified by Central Varietal Release and Notification Committee (CVRC) of India. These 375 varieties includes 5 landrace 369 modern varieties and one hybrid variety (KRH-2)representing five regions of India where rice is grown as a major crop (Table S1). For comparing the efficiency of SSR and SNP markers in assessing genetic diversity and population structure, equal number of locus (thirty-six primers each) of SSR and SNP have been used and compared at the statistical, genetic relatedness and population structure level.

### Statistical Comparison of HvSSR and SNP Markers

**Figure 1:**
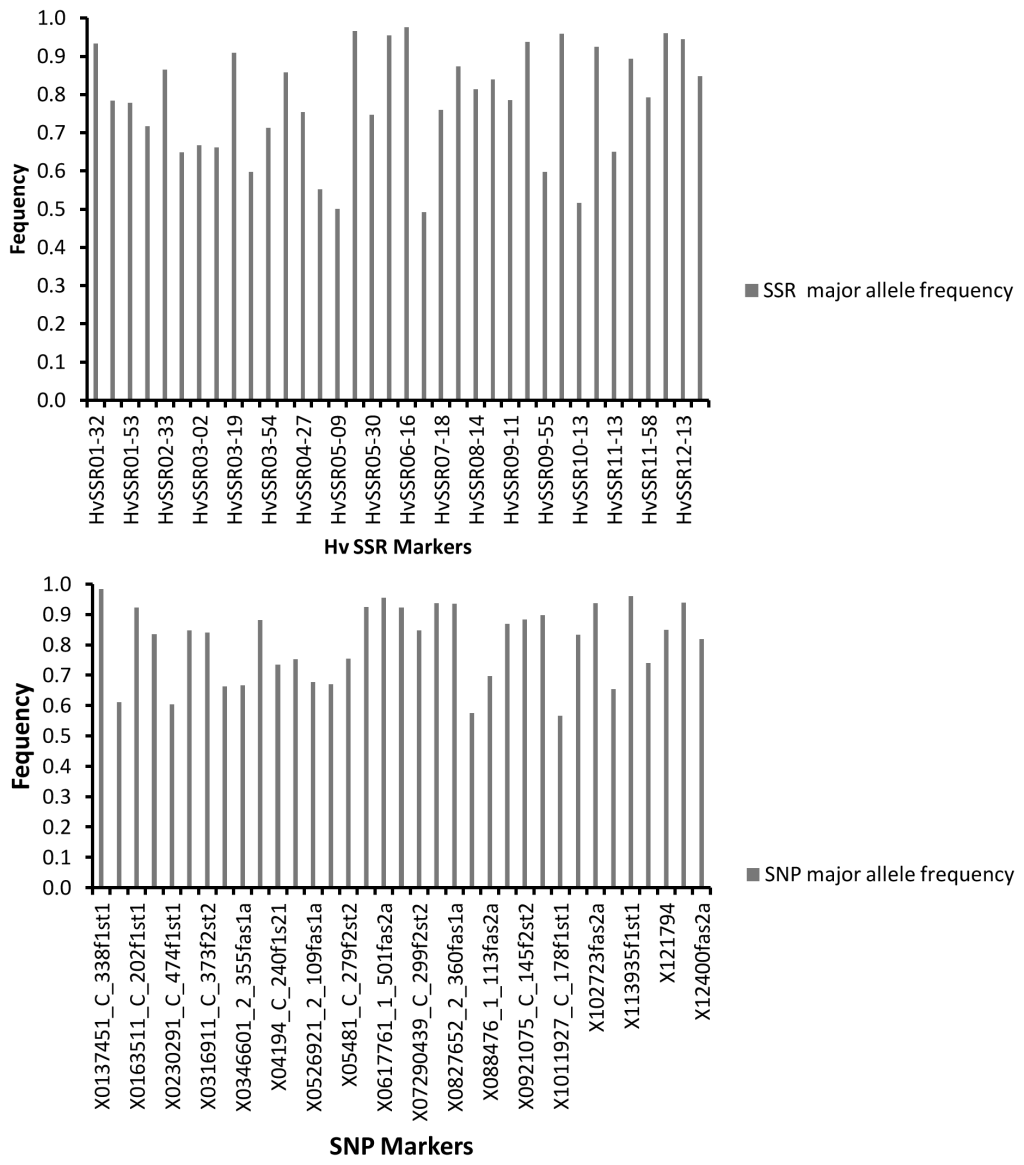
Major allele frequency spectrum for (a) 36 SSR and (b) 36 SNPs in 375 rice varieties.

Temperature of amplification (Ta) for 36 HvSSR primers ranged from 51.9°C to 61.3°C, and used for generating amplification profiles of rice varieties. The number of alleles amplified per SSR primers varied from 2 to 4 (Table 1). Maximum numbers of alleles were amplified by primer HvSSR12-39 (4 alleles). A total of 80 alleles were amplified with an average of 2.22 alleles per locus in 375 varieties. PIC value for HvSSR primers ranged from 0.04 for HvSSR06-16 to 0.5 for HvSSR05-09 with an average of 0.25. The gene diversity ranged from 0.05 to 0.58 with an average of 0.3. Heterozygosity was also calculated and for five loci heterozygosity was zero (HvSSR05-30, HvSSR06-16, HvSSR08-14, HvSSR09-26 and HvSSR10-03). Maximum heterozygosity was present at HvSSR09-55 loci (0.73) and average heterozygosity across all 36 loci was 0.12. The major allele frequency was also calculated for all 36 markers which ranged from 0.49 to 0.97 with an average of 0.78 (Figure 1a, Table 1). 

**Table 1:** List of HvSSR primers used for genotyping of 375 rice accessions along with their chromosomal position, product size, No of alleles amplified, Temperature of Amplification (Ta), Gene diversity, Heterozygosity and PIC value.

**Chr. No.**	**Primer ID**	**Size (bp)**	**Forward primer**	**Reverse primer**	**No of alleles amp.**	**Ann. temp(°C)**	**Gene Div.**	**Heterozygosity**	**PIC**	**Major Allele freq.**
**1**	HvSSR01-32	250	AAACTGGAGATGAACTCGAA	GTAACGAACTAGAGCATGGG	2	55.6	0.13	0.02	0.12	0.93
	HvSSR01-41	348	TGAGTGAGACTTGACAGTGC	AGTTAACACCAATGCTGACC	2	59	0.34	0.005	0.28	0.78
	HvSSR01-53	274	TGTCGTCCACGTAGTAGGAG	ACACTCCTCCTCTGTTCTCA	2	51.9	0.35	0.006	0.29	0.77
**2**	HvSSR02-01	312	AAGAGATGAGAAGAGCAATGA	CAACTTAGAGGAAGAAGGAGG	2	60	0.41	0.18	0.32	0.72
	HvSSR02-33	355	TAATGCACGCACAACTTTAC	TATAGAATGCTGACTGGGCT	2	60.1	0.24	0.03	0.21	0.87
	HvSSR02-50	195	TTTCAGGAATCTGATGCTTT	TTAATCAAAGCCCTAACAGC	2	52	0.45	0.31	0.35	0.64
**3**	HvSSR03-02	228	TAGCGGAGTTGGAATAACAC	CTGCACTGCATACCTCATAA	2	55	0.45	0.008	0.35	0.68
	HvSSR03-10	280	GTACACAACGTCACAACAGC	ACTGTGGCATATGTTCGATT	2	55	0.45	0.26	0.35	0.66
	HvSSR03-19	230	AATTCAGTTCACGCATTCTT	AGCTGTTCGTCTGCATAGTT	2	61	0.17	0.06	0.15	0.91
	HvSSR03-37	386	GGAAATCGTCAAGAACGTC	TAATTGTATACCACTCCGCC	2	59	0.47	0.09	0.36	0.59
	HvSSR03-54	352	GCCTATCAGGCTATCATCAC	GTGATCGACATTGAGGAGTT	2	59	0.40	0.006	0.32	0.71
**4**	HvSSR04-19	265	TCGTGGAGTATCCTGTATCC	TTATAACTTGGAGCTCAGGC	2	56	0.24	0.19	0.21	0.86
	HvSSR04-27	318	ATGGATTTAGGCTTGTTTGA	ATACTGCGAAGGTGAAGAGA	2	58	0.37	0.28	0.30	0.75
	HvSSR04-46	179	GGCGCGCTTATATATGTACT	CGATTGCGTGGTGTAACTAT	2	61.2	0.49	0.03	0.37	0.55
**5**	HvSSR05-09	335	CTCTCCATCTTGCAATCTTC	TGCATGACTCTATCAACCAG	3	61	0.58	0.12	0.50	0.50
	HvSSR05-15	275	CCATGTCAAACGGTTACTTT	GGGAGAAGTGAGAAAGAGGT	2	60.5	0.07	0.01	0.07	0.97
	HvSSR05-30	353	TACGACGGACGATTAAAGTT	GCTAACTCATTCATCTCGCT	2	60.5	0.38	0.0	0.31	0.74
**6**	HvSSR06-03	212	CTAGGGAATCAGCGGTTAG	GCTCTCTTGTCCTTCTTCTTC	2	57.8	0.08	0.01	0.08	0.96
	HvSSR06-16	368	TCTGAAATGCTGTCATCAAG	GAGCAGAGTAGGACATGAGC	2	55.2	0.05	0.0	0.04	0.97
	HvSSR06-40	385	CTCTTCCGTGGTTAAAGAAA	CACTGGTATGATCTCCGACT	3	61	0.52	0.39	0.41	0.49
**7**	HvSSR07-18	343	GGTGTGTTGTCGAATCTCTC	ATGCCATTGTCCTTACATTC	3	61.3	0.39	0.23	0.36	0.76
	HvSSR07-51	341	CGAGCATGTCTGTCAAGTAA	GTTCGAATGTAATGTTGGCT	3	56.3	0.22	0.04	0.21	0.87
**8**	HvSSR08-14	295	TCCACTTTACATCGTCACAA	CTACCTCTTAACCGCACATT	2	59.3	0.30	0.0	0.26	0.81
	HvSSR08-19	221	CATCTCTTGAGAAATCTGCC	TGTGCATTTCGTCTTTCATA	2	55.6	0.27	0.06	0.24	0.84
**9**	HvSSR09-11	366	TGCAGAATTTCTTCCTTCAT	ACCAGAATCTCCCAAATGTA	3	61	0.36	0.01	0.33	0.78
	HvSSR09-26	331	TGGGCATCTGGTACTATCTT	AGCTCATTCCACAGGTTAGA	2	60	0.11	0.0	0.11	0.93
	HvSSR09-55	382	TTACTCCGCATATATCCATGT	ATTTGACACCAAGTTGATCC	2	61	0.48	0.73	0.37	0.59
**10**	HvSSR10-03	289	TCTTTCCCAAATTCCAGATA	CATTAGTTGTTTGTGGCAGA	2	56.3	0.07	0.0	0.08	0.96
	HvSSR10-13	169	CAGGGAATCAACATCAAAGT	AGCAAGGCAAGTCATCTCTA	2	59.4	0.49	0.52	0.37	0.51
	HvSSR10-34	202	TAGACCGAGGAATTGAAAGA	TTTGGGCTTATTGTCAGTTT	2	50.8	0.14	0.07	0.13	0.93
**11**	HvSSR11-13	285	TGAAACCACAATGAGTCAAA	GCCCTAAACCCAAATAGAAG	2	54.1	0.46	0.13	0.35	0.65
	HvSSR11-21	291	TACGCTATAACCATGAAGCA	CTCCCGTTATTGTCCTTACA	3	61	0.19	0.08	0.18	0.89
	HvSSR11-58	371	ACTGAATCCTTACTGGAGCA	GGAGATAAGCATTTGGAAGA	2	54.1	0.32	0.19	0.27	0.79
**12**	HvSSR12-01	271	GATTTGCAACACGTACGATA	GATCATCCACTCTGAGCAAT	2	52.6	0.07	0.04	0.07	0.96
	HvSSR12-13	388	ACCTTAGGGCTGAGTTCTTT	TTAGGCTTGTCTCTTCCTCA	2	60	0.10	0.03	0.10	0.95
	HvSSR12-39	289	ATCTAACAACAACAATCCCG	CATCTTCATCCCTCGTGTAT	4	60.5	0.27	0.13	0.26	0.85
				**Mean**			**0.30**	**0.12**	**0.25**	**0.78**

The unlinked SNP markers located on the short arm, centromeric region and long arm of all 12 rice chromosomes were developed and used for diversity analysis. The chromosome number, primer ID and their physical position on rice chromosome are given in Table 2. SNP is bi-allelic in nature therefore; total 72 alleles were amplified with an average of 2 alleles per locus in all tested rice varieties and the major allele frequency ranged from 0.56 to 0.98 with an average of 0.80 (Figure 1b, Table 2). Alleles were scored as wild, heterozygous and alternate alleles across all the 375 rice varieties and the percent allele was calculated for all 36 loci (Table 2). Highest heterozygous alleles (58.6%) were found with marker 11-1849 located on chromosome 11. The PIC value was highest for primers 08-4218-5_C_129 and 1011927_C_178 (0.37) and lowest for primer 01-3916-1_C_156 (0.03) with an average PIC value of 0.23. The gene diversity also ranged from 0.03 to 0.49 with average gene diversity 0.28. Heterozygosity across the 36 loci ranged from 0.02 to 0.64 with mean value of 0.19.

**Table 2:** List of SNP primers used for genotyping of 382 rice accessions along with base call, gene diversity, heterozygosity and PIC.

**Chr. No.**	**Primer ID**	**Physical position**	**Amplification primer1**	**Amplification primer2**	**Allele percentage**			**Gene Diversity**	**Heterozygosity**	**PIC**	**Major Allele freq.**
					**Wild**	**Het**	**Alternate**				
**1**	01-3916-1_C_156	25381654	ACGTTGGATGGGGTTTGCATGTTAATAGGG	ACGTTGGATGCCGAATCTCTATCAAGGAAG	87	3	0.0	0.03	0.03	0.03	0.98
	01-608-4_C_375	3421011	ACGTTGGATGAGGACCATCTTCTTGCACTG	ACGTTGGATGCCATTTGCAAGGCCCATTTC	42	33.0	21	0.47	0.34	0.36	0.61
	01-6351-1_C_202	40914292	ACGTTGGATGGTTGGAACACATGATTTCAC	ACGTTGGATGATCTCTTTGGACAGAGTCCC	72	7	3	0.14	0.08	0.13	0.92
**2**	02-267	1570149	ACGTTGGATGGTCAATCTTGCAGGAGTTGG	ACGTTGGATGTGGCTCCTCTTCTCCGGTCT	68	17	7	0.27	0.18	0.24	0.84
	02-3029-1_C_474	18821156	ACGTTGGATGTGTCTGCAATAACTTGTGCC	ACGTTGGATGAAATCAGCTGCAGCATTACC	38	26	21	0.47	0.30	0.36	0.60
	02-4333-1_C_293	28688819	ACGTTGGATGGGAATGTTTAGTTTTGAGG	ACGTTGGATGTGTAGGTGCTACTTGCTTCC	53	10	5	0.25	0.14	0.22	0.85
**3**	03-1691-1_C_373	10849512	ACGTTGGATGAACAACGCCAGGAACATCAC	ACGTTGGATGAAGCGGCTCAAGGTACAATC	58	13	6.0	0.26	0.16	0.23	0.84
	03-3478-1_C_206	22815422	ACGTTGGATGCCTGCAGCAAACGCCAATTT	ACGTTGGATGTCAGGTAACCGATCGATTTG	6.0	40	31	0.44	0.51	0.35	0.66
	03-4660-1_C_355	31020366	ACGTTGGATGCTCCCATCCTAGTATCCATC	ACGTTGGATGTGCCTTCTCTTACAGGTTCC	40	14	17	0.45	0.19	0.35	0.67
**4**	04-1801-20_C_428	11859836	ACGTTGGATGCCCTCAAAAAAAAGTTGTAAG	ACGTTGGATGCAGTAAATTTCCAGGGAGATA	75	23	0.0	0.21	0.23	0.18	0.88
	04-19-4_C_240	225838	ACGTTGGATGTCTACACATTAGCTCGCTGG	ACGTTGGATGACAGTAACCACAATATGCCG	8	5.0	28.0	0.39	0.12	0.31	0.73
	04-3787-3_C_358	25211800	ACGTTGGATGTTATCTCTGCTTGCTCGCTC	ACGTTGGATGAAGTATCTGCCCCAAGTGAC	43	33	3	0.37	0.41	0.30	0.75
**5**	05-2692-1_C_109	18783426	ACGTTGGATGGAACTTTACTCTCAGTACA	ACGTTGGATGTGGTTTGATGAGTCGTTTGC	31	27	8	0.43	0.41	0.34	0.68
	05-4192-1_C_280	28065769	ACGTTGGATGAGTTTGTTGACAGCAGAACC	ACGTTGGATGTAGCTTACTAGTTCATGTG	28	9	11.0	0.44	0.19	0.34	0.67
	05-48-1_C_279	287362	ACGTTGGATGCAGAGATGTCTGTTGTTAGC	ACGTTGGATGCAACCAGGGATACAATATGAC	34	11.0	7	0.37	0.21	0.30	0.76
**6**	06-1256-1_C_147	7573979	ACGTTGGATGCACGTGCCTATGATTAGCAG	ACGTTGGATGGATCGTTTACTTCTTTGCCC	85	6	4	0.14	0.06	0.13	0.92
	06-1776-1_C_501	11093772	ACGTTGGATGGGGCCAATTTGCTTAGTGC	ACGTTGGATGAGCATAAGGTATTAAAGTC	62	2	2	0.08	0.03	0.08	0.96
	06-2509-1_C_497	15737387	ACGTTGGATGCCTTCGCGCTTGCAATTTGG	ACGTTGGATGAAATCAGCACGCGTCAACAC	49	3	2	0.14	0.06	0.13	0.92
**7**	07-2904-39_C_299	19160255	ACGTTGGATGAATGGTGGTGTATCTTGAGC	ACGTTGGATGGGTGTGACTTCTCATGACAG	65	20	3	0.26	0.22	0.23	0.85
	07-293-12_C_368	1859603	ACGTTGGATGCACTAATTCTTGGTATTATGG	ACGTTGGATGTCAATGTGTTCTCACAGACC	27	1	2	0.12	0.02	0.11	0.94
**8**	08-2765-2_C_360	18084851	ACGTTGGATGTCCCTCCATGTTGTGAGTTC	ACGTTGGATGCTTGCAAGAGACATCCAAGA	94	0	0	0.12	0.03	0.11	0.94
	08-4218-5_C_129	27692470	ACGTTGGATGGGTGGACAAAGATAAGGAAG	ACGTTGGATGGACTGGAAATATACTCCCTC	25	1	1	0.49	0.26	0.37	0.57
	08-847-6_C_113	5399913	ACGTTGGATGCCCAACGTATTAATGGCAAC	ACGTTGGATGGCTGTGTAGTAATTTGCCTG	18	11	12	0.42	0.05	0.33	0.69
**9**	09-209	1297966	ACGTTGGATGGAGGCAAAAGGCAAACCGAC	ACGTTGGATGGACTTGAGCGAGTCGATGTC	23	2	10	0.23	0.22	0.20	0.87
	09-2107-5_C_145	13705487	ACGTTGGATGTGACCACACCACACAAACAC	ACGTTGGATGGGGATTTGCGGTTTTTGGAC	66	20	2	0.20	0.19	0.18	0.88
	09-2716-4_C_457	19541336	ACGTTGGATGTGAGCCACAGATTCCCTTTC	ACGTTGGATGCTCGAGTAATTCAAAACCAC	67	17	2	0.19	0.07	0.17	0.89
**10**	10-1192-7_C_178	8122635	ACGTTGGATGCTTTGCTACGGATAAAATG	ACGTTGGATGTCATGCAAATACAGACATGG	61	5	5	0.49	0.36	0.37	0.57
	10-188-1	1218215	ACGTTGGATGGCGCCAGTGTATGGAAAAAG	ACGTTGGATGGTCCATAACATCATGGACTC	22	21	14	0.28	0.27	0.23	0.83
	10-2723	20696970	ACGTTGGATGCCCACAATGAGATGCAGATG	ACGTTGGATGAGACAAAATGCAACACTCCG	53	21	2	0.12	0.13	0.11	0.94
**11**	11-1849	11974790	ACGTTGGATGCGCCACTCTTCCTGATTTAG	ACGTTGGATGACAGATACGGGAGGCATTTC	67	10	0	0.45	0.64	0.35	0.65
	11-3935	28434679	ACGTTGGATGATCCCTGAGACTTTGGATGG	ACGTTGGATGCCAACTTGAATGTCCATTCC	31	59	2	0.07	0.03	0.07	0.96
	11-522-1_C_214	3033366	ACGTTGGATGCTACATGGTATCAGATACCG	ACGTTGGATGAGAAGCGAACGCGGAAAAAG	79	3	2	0.38	0.23	0.31	0.74
**12**	12-1794	11215946	ACGTTGGATGGTGAGCCCCAAAAGTTGGTG	ACGTTGGATGTAAGGTCCAGTTTGCTTGGT	54	20	12	0.26	0.11	0.22	0.85
	12-3200-2_C_389	21396181	ACGTTGGATGGCTCAAACCTAGCAATAACTG	ACGTTGGATGCCTCCTTCCTACAAGTTTAA	77	11	9	0.11	0.02	0.10	0.94
	12-400	2160546	ACGTTGGATGCCAATAGAGTCCATCTCAGC	ACGTTGGATGGCACGAGGATTTAAGACAGC	25	1	1	0.29	0.33	0.25	0.82
				**Mean**	50	26	1	**0.28**	**0.19**	**0.23**	**0.81**

### Comparison of HvSSR and SNP Markers in Genetic Relatedness Study

**Figure 2:**
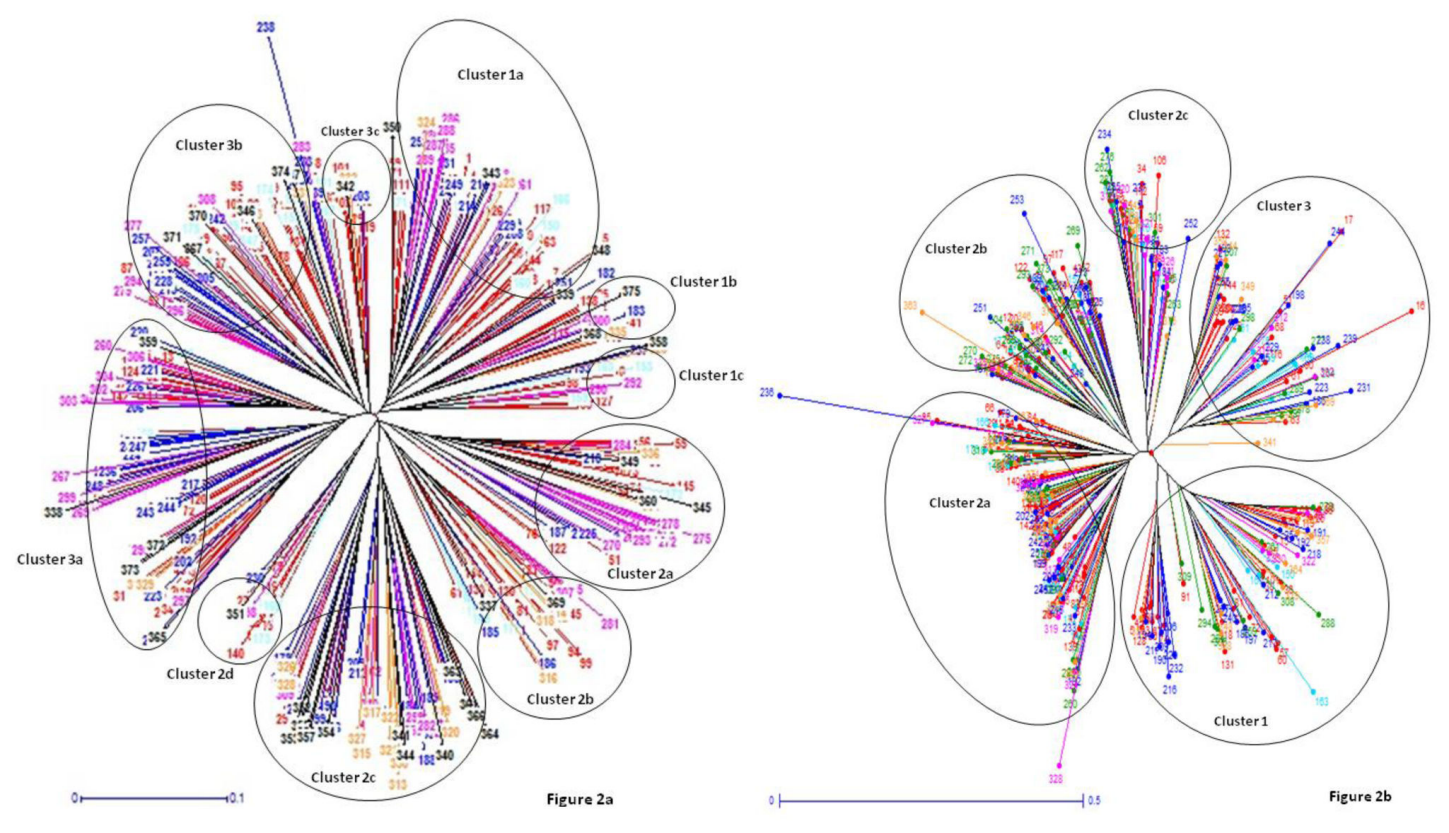
NJ tree constructed for (a) SSR and (b) SNP data based on regions south (red), north-east (sky blue), east (blue), north (green), west (pink) and CVR (orange).

All the HvSSR amplicons generated across 375 varieties were assessed for genetic distance and the dissimilarity matrix was used for cluster development using the neighbour joining (NJ) method. In the Unrooted tree (Figure 2a) and Phylogram (Fig. not shown) rice genotypes were grouped into three major clusters. Further cluster1 was sub-grouped into cluster1a which contains 66 varieties, cluster1b with 13 varieties and cluster1c with 14 varieties. Similarly, cluster2 was sub-grouped into four clusters; cluster2a, cluster2b, cluster2c and cluster2d with 37, 29, 56 and 12 varieties respectively. Cluster3 was the largest containing 52 varieties in cluster3a, 87 varieties in cluster3b and 9 varieties in cluster3c. Grouping of the rice varieties labeled with different colours to represent different regions were getting represented in all the major clusters and even in the sub clusters. Further, traits of some of these varieties as recorded in the passport data and their grouping into different clusters was analyzed to find trait based grouping (if any). Interestingly in the cluster1c and 2d varieties having resistance to blast disease were grouped (Table S2).

Similarly all 36 SNP loci were scored across all the tested rice varieties. Genetic distance was calculated and NJ tree was constructed using dissimilarity matrix. Unrooted tree (Figure 2a) as well as Phylogram (Fig. not shown) was generated to find genetic relationships among the rice varieties. All varieties were grouped into three major clusters. Cluster2 which was the major cluster further subdivided in to three sub clusters2a, 2b and 2c, respectively (Figure 2b). In the cluster2a 127 varieties, cluster2b 76 varieties cluster 2c 43 varieties were grouped whereas, in cluster1 and cluster3, 75 and 54 varieties grouped, respectively. Grouping pattern of the rice varieties using SNP markers were found similar to SSRs. However, in cluster 2a out of 127 varieties 24 varieties were from Kerala which could be a notable observation regarding geographical isolation. No significant grouping was observed for trait on the basis of information available as passport data.

**Figure 3:**
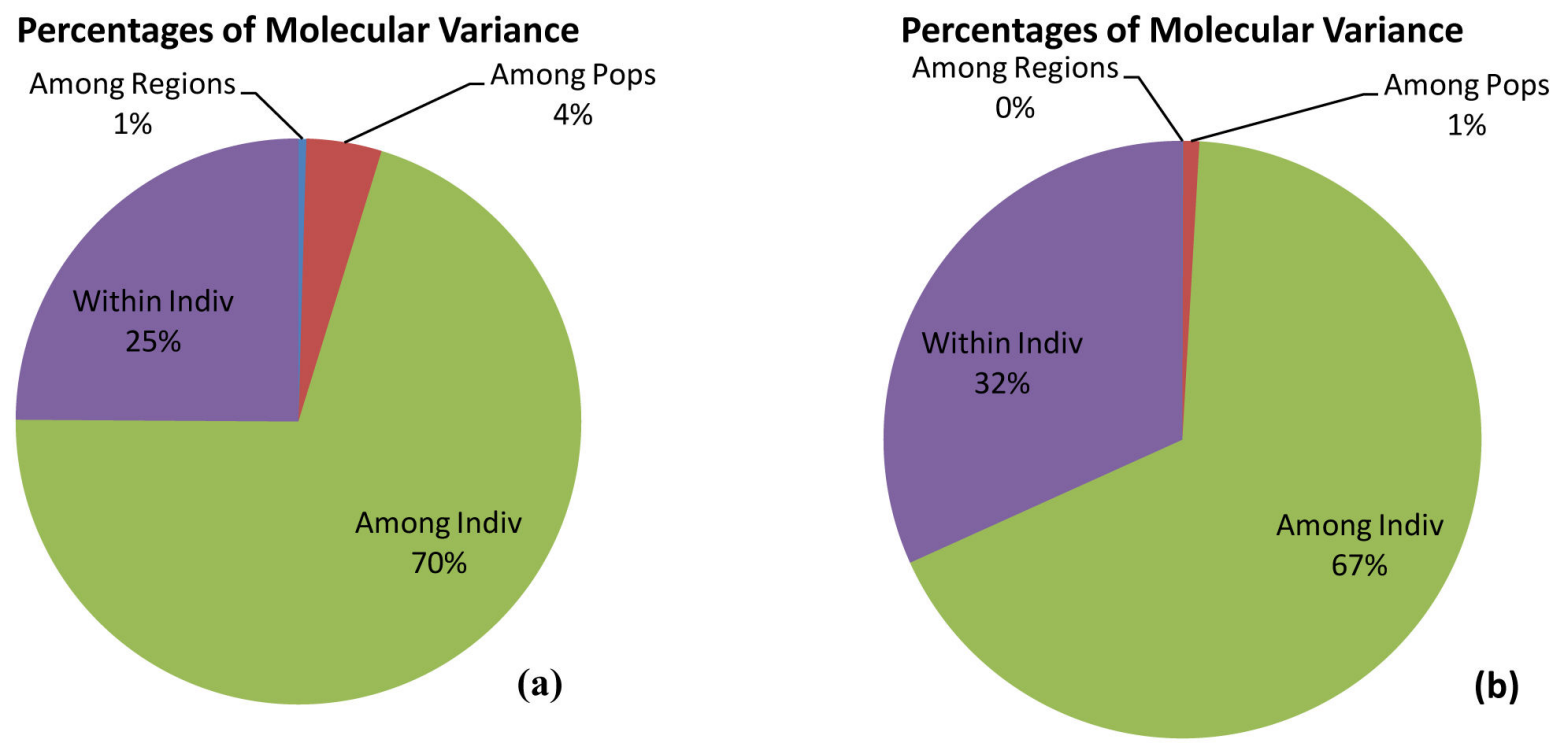
Analysis of Molecular variance (AMOVA) of 375 rice varieties based on (a) SSR data and (b) SNP data.

Analysis of Molecular Variance (AMOVA) with HvSSR showed that among regions only 1% diversity existed whereas at population level it was 4%. Maximum diversity has been observed at individual’s level (70 %) and within individual (25%) level (Table 3, Figure 3a). In case of SNP maskers among regions, no diversity exists whereas at population level 1% diversity existed (Table 3, Figure 3b) and maximum diversity was partitioned between individual level (67 %) and within individuals (32%). 

**Table 3:** Summary AMOVA for SSR and SNP markers.

**Source**	**AMOVA for SSR**					**AMOVA for SNP**			
	**df**	**SS**	**MS**	**Est. Var.**	**%**	**SS**	**MS**	**Est. Var.**	**%**
**Among Regions**	5	171.207	34.241	0.035	1%	90.579	18.116	0.003	0%
**Among Populations**	12	297.559	24.797	0.335	4%	198.662	16.555	0.073	1%
**Among Individual**	357	4600.038	12.885	5.474	70%	4979.079	13.947	5.643	67%
**Within Individual**	375	726.500	1.937	1.937	25%	998.000	2.661	2.661	32%
**Total**	**749**	**5795.304**		**7.781**	**100%**	**6266.320**		**8.381**	**100%**

**Figure 4:**
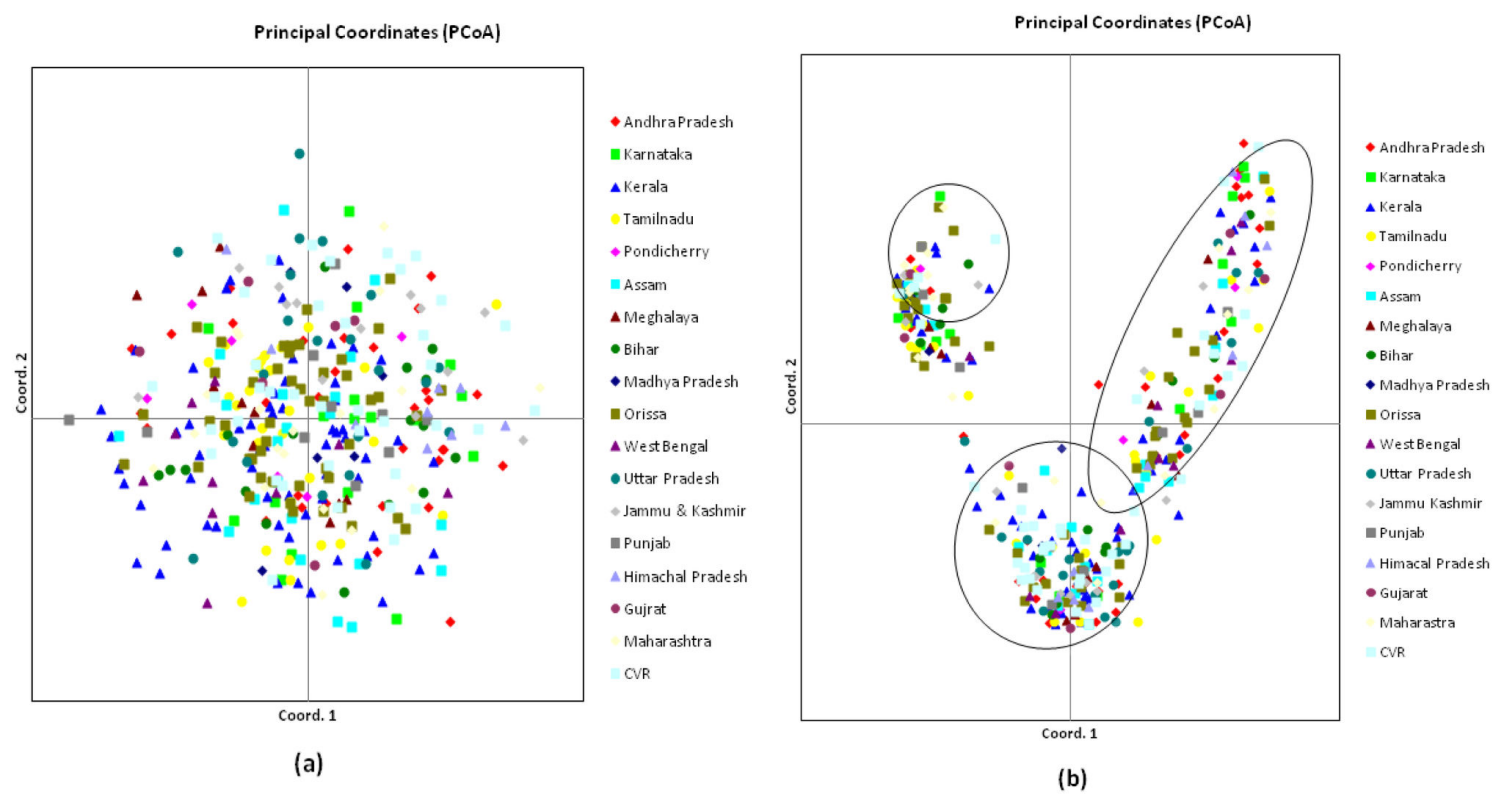
Principal Coordinate Analysis (PCoA) of 375 rice varieties based on (a) SSR data and (b) SNP data.

Principal Coordinate Analysis (PCoA) with SSR markers showed that large diversity existed in Indian rice varieties. Varieties exhibited uniform distribution across the two axes (Figure 4a). The first three axes explained 13.33% of cumulative variation (Table 4). In PCoA all varieties were labeled with different colours based on their different regions to indicate their region specificity (Figure 4a), the intermixing of colour across the coordinates further, support the unrooted tree that there is no location-specific grouping of the samples because all varieties were intermixed across the coordinates whereas, in case of SNP markers varieties were getting grouped into three broad groups across the first two axes (Figure 4b). The first three axes of SNP explained 45.20% of cumulative variation (Table 4). In PCoA, the intermixing of colour across the coordinates, further support the unrooted tree with SNP marker that, there is no location-specific grouping. 

**Table 4:** Percentage of variation explained by the first 3 axes using SSR and SNP markers in Principal Coordinate analysis.

	**SSR Markers**			**SNP Markers**		
**Axis**	**1**	**2**	**3**	**1**	**2**	**3**
**%**	4.72	4.52	4.08	27.07	9.40	8.73
**Cum %**	4.72	9.25	**13.33**	27.07	36.47	**45.20**

In present study 29 Autumn rice varieties, also known as '*Aus*' in West Bengal were compared with *indica* subpopulations and based on AMOVA, 1% variation (Table S3a, and Figure S1a) with Fst value 0.009 (Table S3b) was found between the two subpopulations based on SSR markers but no such population difference was observed with SNP markers (Table S4a & S4b, Figure S1b). 

### Comparison of HvSSR and SNP Markers in Population Structure Study

**Figure 5:**
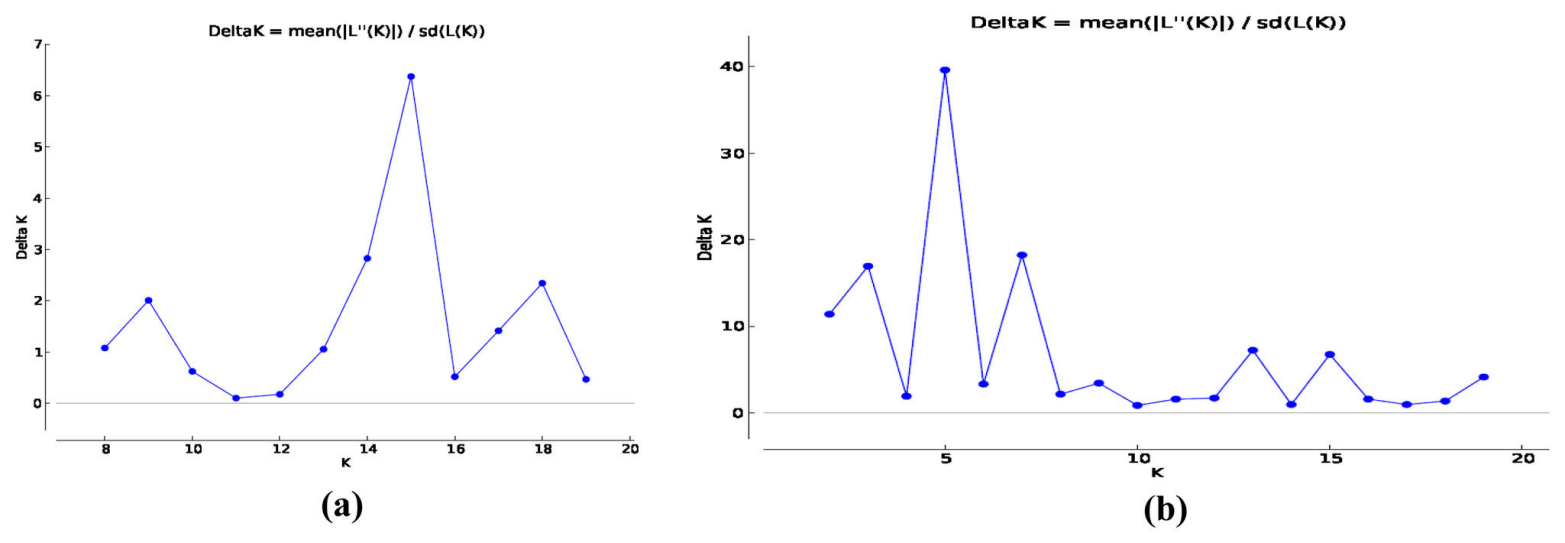
Estimation of population using LnP(D) derived Δk for k from 1 to20, (a) SSRs and (b) SNPs.

**Figure 6:**
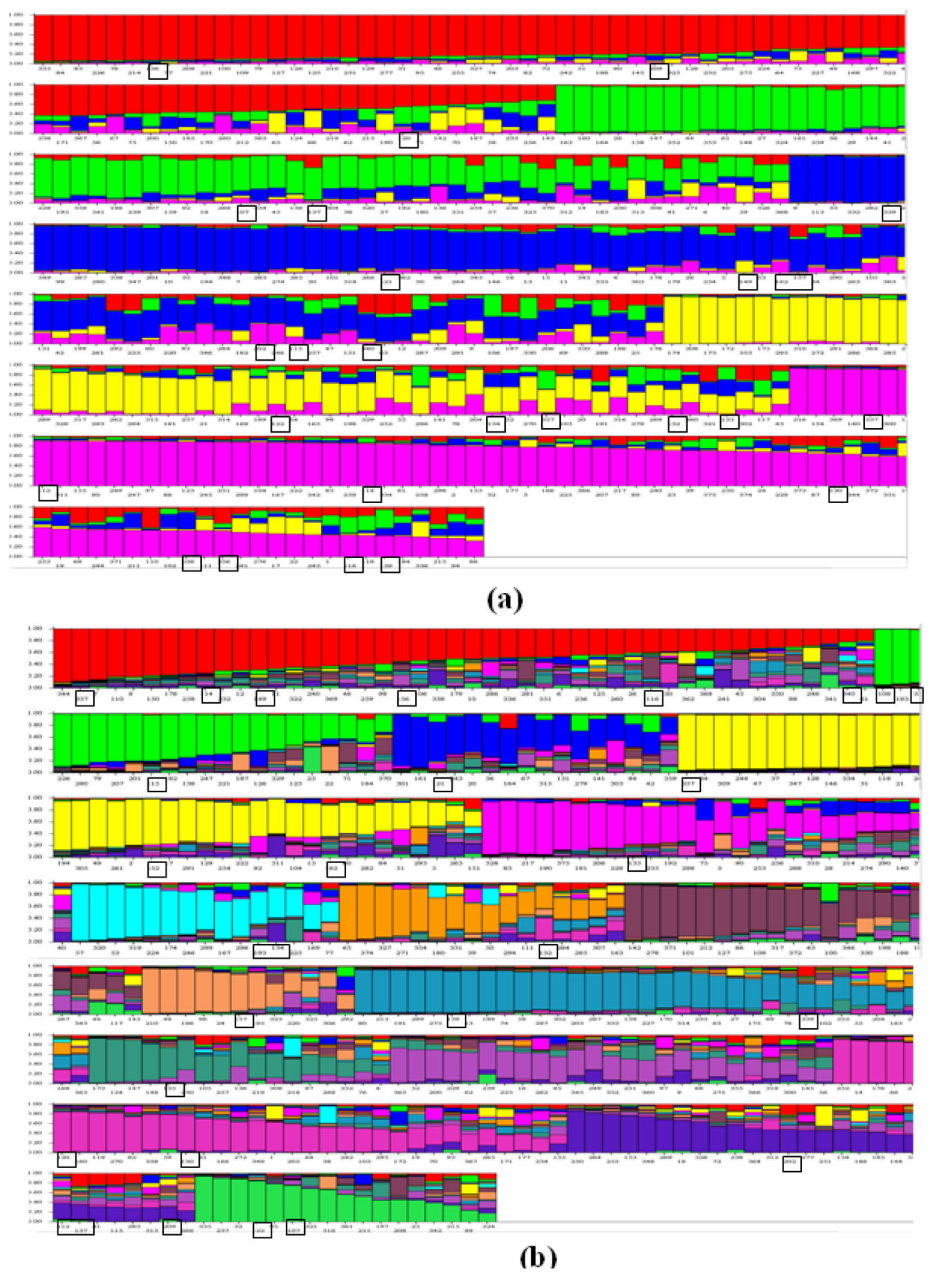
Model based clustering of (a) SSR (K=5) and (b) SNP (K=15) of Indica rice cultivars, (boxes indicates distribution of Autumn rice, also known as 'Aus' in West Bengal, 'Ahu' in Assam, 'Beali' in Orissa, 'Bhadai' in Bihar, 'Virippu' in Kerala and 'Kuruvai/kar/ Sornavari' in Tamil Nadu).

To study the population structure, a model based programme STRUCTURE was used to determine genetic relationship among individual rice varieties. This model assumes that the number of populations was k and the loci were independent and at Hardy–Weinberg equilibrium. In the case of SSR, K=1 to K=20 population were tested, Ln(PD) kept on increasing with increasing population number but there was no clear population structure, therefore Ln(PD) derived Δk was plotted against the K to determine the number of populations using a software “Structure harvester” available online. At K=5 maximum Δk was found (Figure 5a) and this was considered as number of population for 375 Indian rice varieties. In population1 79 varieties, population2 63 varieties, population3 93 varieties, population4 57 varieties and in population5 83 varieties were grouped. Further, using structure analysis (Figure 6a) varieties under different populations were categorised as pure or admixture and for categorisation purpose varieties with more than 0.80 score were considered as pure and less than 0.80 as admixture. In population1 39 pure and 40 admix, population2 29 pure and 34 admixture population3 42 pure and 51 admixture, in population4 19 pure and 38 admixture and in population5 31pure and 52 admixture were present and in total 215 pure and 160 admixture were identified (Figure 6a).

Similarly in case of SNP markers Δk values were plotted against the K to determine the number of populations and at K=15maximum Δk was found (Figure 5b) Further, K=15 number was closer to number actual number of Indian states considered for the present study. Pure and Admixture in two populations were decided as above in case of SSR markers. In population K=15, 66 pure and 309 admix were present (Figure 6b). Observation of only 66 pure individuals in the K=15 population is further supported by the pedigree because only few varieties were developed using local material whereas majority for modern varieties were bred using genetic material introduced from different locations (Table S1).

The grouping of *Aus* and *Indica* subpopulation was also analysed with both SSR and SNP markers but no specific grouping of *Aus* was observed from *indica* subpopulation (Figure 6a & 6b).

## Discussion

Molecular basis of polymorphism and their distribution across the genome is quite different for SNP and SSR markers. Hence, the utility of SSR/ SNP marker in crop improvement will depend on the quality of information they provide with respect to parameters for genetic diversity and population structure. This is the first such study where SSR and SNP marker system were assessed for their efficiency in assessing genetic diversity and population structure in the large collection of Indian rice varieties. This study is also important on the account that India is the centre of origin for rice and varieties released for cultivation in five major regions are expected to be diverse because they are released by different states according to their suitability to respective agro-climatic conditions. A comprehensive analysis of SSR and SNP markers in such a diverse set of varieties may assist rice breeders in deciding which one of the two is appropriate for the rice germplasm characterization and designing breeding strategies. 

The SSR vs SNP comparison in Indian rice collection for statistical parameters, such as number of alleles amplified per primers (2.2/2.0 allele per primer), gene diversity (0.30/0.28), heterozygosity (0.12/0.19) and PIC value (0.25/0.23), revealed that both marker systems generated almost similar information when equal number of locus (36 locus each) were studied. Earlier reports say that more SNP loci should be studied when comparing diversity generated by SSR [26]. Further, comparison of our findings with previous studies in rice using both SSR and SNP markers, showed mixed results in number of cases; low numbers of alleles were reported for SSR markers. The low number of alleles recorded in this study may be mainly due to the resolution effectiveness of the metaphor agarose used for separation of amplified products. Various studies in rice using agarose/ PAGE gels have reported lower numbers of alleles [27-29]. Another reason for reduced number of alleles may be the exclusion of monomorphic and spurious bands from analysis. The low average PIC value (0.25) observed with SSR markers indicates lower genetic diversity among the rice varieties considered for our study. The high average PIC value for SSRs have been mainly reported with diverse germplasm lines because they are genetically more diverse compared to varieties. PIC depends upon many factors such as breeding behavior of the species, genetic diversity in the collection, size of the collection, sensitivity of genotyping method and location of primers in the genome used for study. Low PIC value with SSR markers was found in other rice collection studies [29,30]. For the SNP markers PIC values ranged from 0.03 to 0.37 with an average PIC value 0.23. PIC value in the similar range was reported for SNP markers in a collection of rice varieties by Chen et al [31]. Moreover, due to bi-allelic nature of SNPs, their PIC values can range from 0 to 0.5. Whereas for SSR markers which are mutli-allelic PIC value goes above 0.5 and it can go up to 1.0. Therefore, in our study, SNP markers with mean PIC value 0.23 were more informative than SSR markers with mean value of just 0.25. 

Furthermore, efficiency of both the markers was compared in assessing genetic relatedness among the varieties on the basis of their clustering pattern in the unrooted tree. Although the unrooted trees constructed using either SSR or SNP markers, grouped varieties into three major clusters, the numbers of varieties grouped into the clusters were different for both the trees. These findings were not surprising as broad pattern of grouping is expected to be more or less similar irrespective of type of markers used for genetic relatedness study. Similar, findings were also reported in European rice collection by Courtois et al. [17]. Traits available in passport data were analyzed to find out trait based grouping and in case of HvSSR in cluster 1c and 2d some of the varieties possessing resistance against blast were grouped together. No geographical isolation has been observed in clusters except in case of SNP tree where in cluster2a out of 127 varieties 24 varieties were from Kerala could be a significant observation regarding geographical isolation. Lack of geographical isolation observed in the present study was mainly due to frequent introduction of genetic material from different locations for development of modern rice varieties. AMOVA analysis also indicated that at regions only 1% diversity exists whereas among population level 4% diversity exists in HvSSR and 1% at population level in SNP with the both the markers maximum diversity is getting portioned at individual’s level (70 % in case of SSR and 67% in case of SNP) and within individual (25% in SSR and 32% in SNP) level. AMOVA study shows that HvSSR marker has explained better partitioning of variation than SNP markers. Similar partitioning of variation at population and sub population level has been reported in case of rice [32]. The PCoA plot analysis of rice varieties using SSR vs SNP generated interesting results. Though, the broad pattern of distribution of varieties in the PCoA plots was similar with both the markers, but a closer look revealed three major clusters for rice varieties in case of SNP markers, such grouping was not found in case of SSR markers. Similar observation has been reported in case of wheat with SNP markers [33]. The proportion of variance explained by first three coordinates in case of SNP (45.20%) was higher than the SSR (13.33%) which in accordance with finding in maize [34] and Wheat [33]. Overall at the genetic relatedness level SSR markers are more informative as compared to SNP markers. Therefore, it may be concluded on the basis of this study that for genetic diversity analysis HvSSR markers were more effective. SSR marker in genetic diversity analyses have been a powerful tool because these markers are neutral, multi-allelic and co-dominant in nature (Lapitan et al. 2007)[27]. 

Based on SSR and SNP markers study *Indica* rice has been subdivided into two subpopulations *Aus* and *Indica* [3,35,36]. In the present study SSR markers support the grouping but with SNP marker no such clear distinction between the two subpopulations was observed. There are three seasons for growing rice in India viz.- autumn, winter and summer. Autumn rice is known as 'Aus' in West Bengal, 'Ahu' in Assam, 'Beali' in Orissa, 'Bhadai' in Bihar, 'Virippu' in Kerala and 'Kuruvai/kar/ Sornavari' in Tamil Nadu. The pedigree of modern varieties also shows that autumn rice has been frequently used as one of the parent in the development of modern varieties, such as Ratna, Manoharsali and Annada (Table S1) has been used for development of Shanti, Himalaya-2, Nagarjuna, Chandan, Kapilee and Luit. This may be another reason for less distinction between the two subpopulations. Similar trend was observed with population structure where *Aus* did not show distinct grouping from *indica* subpopulation (Figure 6a & 6b).

At population level, no clear population structure for the rice varieties was observed either with SSR or SNP markers which may be due to large genetic variation or frequent intermixing of rice varieties in rice crossing programme across the regions. Therefore ad hoc population was determined using Δk and different populations numbers were found with SSR (K=5) and SNP (K=15) markers. The genetic structures of populations have been previously reported in rice [37-41]. The population observed in these studies ranged from K=3 (Zhao et al. 2009) to K=7 (Jin et al. 2010). The higher value (K = 7) is primarily due to the higher number and diverse set of germplasm [41]. The present study generated a population structure with five clusters using SSR, and fifteen clusters using SNP. Fifteen population cluster and large number of admixture varieties with SNP indicated that population structure can be better explained with SNP markers, because in released varieties of Indian rice to create variation large number of diverse parents has been used. Since admixture is the representation of diverse parents, which themselves have diverse ancestry in breeding history and domestication, may be main reason for variation present in the population [42]. Since, in the SNP based population structure rice varieties appeared subdivided in more clusters than SSR, indicated ability of SNP marker system in delineating population structure at fine level in crops.

In conclusion, though SSR/SNP are multi-allelic/bi-allelic nature and have different distribution pattern over genome even then in the present study equal number of locus (thirty-six each) amplified almost equal number of alleles in case of SSR (80) and SNP (72) markers. However, the unique features of SNP markers such as, abundance in the genome, ability to generate polymorphism due to variation at single base level and their development from the conserved single-copy rice genes [19], enabled these markers to present different diversity spectrum as well as the population structure in Indian rice varieties as compared to the SSR markers. At the population structure level SNP markers showed better genetic relatedness with more population number whereas at the diversity level SSR showed better grouping samples even at trait level. For this reason, SNP markers should be preferably used for determination of population structure in crops. Moreover, SNP markers are mostly derived from genes, as a result genetic diversity assessed using these markers reveals functional variations and may be potentially exploited for the marker trait association studies. Additionally, SNP markers in the present study were derived from the genomic region of rice having synteny with wheat genome and therefore may be equally useful for assessing genetic diversity, population structure and other marker based studies in wheat.

## Supporting Information

Table S1 **List of rice varieties genotyped using SSR and SNP markers.**
(DOCX)

Table S2 **Details of rice samples showed trait based grouping with SSR markers.**
(DOCX)

Table S3 **a.** AOMVA analysis between *Indica* rice population (345 varieties) and *aus* rice population (29 varieties) after removing hybrid rice (1 variety) sample based on SSR marker. **b**. F-statistics analysis between *Indica* rice population (345 varieties) and *aus* rice population (29 varieties) after removing hybrid rice (1 variety) sample based on SSR marker.
(DOCX)

Table S4 **a.** AOMVA analysis between *Indica* rice population (345 varieties) and *aus* rice population (29 varieties) after removing hybrid rice (1 variety) sample based on SNP marker. **b**. F-statistics analysis between *Indica* rice population (345 varieties) and *aus* rice population (29 varieties) after removing hybrid rice (1 variety) sample based on SNP marker.
(DOCX)

Figure S1 **Analysis of Molecular variance (AMOVA) between *Indica* rice population (345 varieties) and *aus* rice population (29 varieties) after removing hybrid rice (1 variety) based on (a) SSR data and (b) SNP data.**
(DOCX)
