# Supplementary material for: Comparison of SSR and SNP Markers in Estimation of Genetic Diversity and Population Structure of Indian Rice Varieties
Source: PLoS One. 2013 Dec 19;8(12):e84136. doi: 10.1371/journal.pone.0084136 (PMC3868579; doi:10.1371/journal.pone.0084136)
Supplement: Table S1 — List of rice varieties genotyped using SSR and SNP markers. (DOCX) [file pone.0084136.s001.docx]

**Table S1** List of rice varieties genotyped using SSR and SNP markers

| **S. No.** | **IC No. ^A^** | **Region** | **^+^State** | **Local Name** | **Pedigree** | **Status*** | **^@^Important Traits** |
| --- | --- | --- | --- | --- | --- | --- | --- |
| 1 | IC140221 | South | AP-5 | Swarna | Vasishtha x Mahsuri | DT | High yielding, low input response areas and resistant to MB and recommended for saline soil |
| 2 | IC140222 | South | AP-14 | Chaitanya | Sowbhagya x ARC-5984 | DT | Resistant to BPH, high yielding, recommended for saline soil |
| 3 | IC140223 | South | AP-17 | Krishnaveni | Sowbhagya x ARC-5984 | DT | High yielding, non-lodging ,resistant to BPH |
| 4 | IC140224 | South | AP-7 | Pratibha | Sowbhagya x ARC-6650 | DT | High yielding |
| 5 | IC140225 | South | AP-2 | Prabhat | IR 8 x MTU 3 | DT | High yielding, BPH resistant variety, grain dormancy strong |
| 6 | IC140226 | South | AP-10 | Vajaram | MTU 4569 x ARC-6650 | DT | High yielding non-lodging |
| 7 | IC248377 | South | AP-18 | Ravi | M-63-83 x (RP-79-5 x Rikutonorin-21)((IET-7991)) | DT | Grains: long bold, resistant to blast, moderately resistant to BS, drought tolerant; Yield: 45 Qxha. |
| 8 | IC296158 | South | AP-27 | Shanti | Ratna x IR-36 | DT | Semi dwarf , long slender grain, resistant to blast, moderately resistant to sheath rot brown spot and WBPH |
| 9 | IC75533 | South | AP-4 | Phalguna | IR-8 x Siam-29 (IET-2911) | DT | Dwarf (86-94 cm), grains: LS, AWA, resistant to GM, moderately resistant to blast and SB, moderately susceptible to BLB and RTV, Yield: 56 Qxha. |
| 10 | IC296246 | South | AP-19 | Krishna Hamsa | Rasi x Fine Gora (IET-9219) | DT | Semi-dwarf, grains: LS, long, fine; Yield: 50-56 Qxha. |
| 11 | IC296322 | South | AP-26 | Nidhi | Sona x ARC-14529 (IET-9994) | DT | Semi-dwarf (95 cm), grains: LS, white and resistant to blast, RTV, tolerant to GLH, WBPH & BPH; Yield: 40 Qxha. |
| 12 | IC308169 | South | AP-15 | Nagarjuna | Sona x Manoharsali (IET-6315) | DT | Semi dwarf, grains: LS, resistant to BPH & blast; Yield: 65 Qxha. |
| 13 | IC308321 | South | AP-29 | Seshu | T-141 x IR-661-34-8 (IET-2881) | DT | Semi dwarf, grains: LS, white, Yield: 41 Qxha. |
| 14 | IC443789 | South | AP-8 | Samba Mahsuri | GEB-24 x T(N)1 x Mahsuri | DT | Resistant to major bacterial blight genes, Semi dwarf, grains: MS, tolerant to blast; Yield: 55-65 Qxha. |
| 15 | IC443790 | South | AP-12 | Saleem | GEB-24 x Sigadis x IR-8 x RNR-8102 (IET-5709) | DT | Semi dwarf (98 cm), grains: LS; Yield: 65 Qxha |
| 16 | IC443791 | South | AP-13 | Satya | Tella Hamsa x Rasi (IET-7720) | DT | Semi dwarf (100 cm), grains: LS, white; Yield: 60 Q xha. |
| 17 | IC443792 | South | AP-16 | Chandan | Sona x Manoharsali (RNR-74802) | DT | Semi dwarf, grains: LS, resistant to BPH, blast & tolerant to GM; Yield: 65 Qxha. |
| 18 | IC443798 | South | AP-30 | Sona Mahsuri | {(GEB 24 x T (N)-1) + Mahsuri} | DT | Aromatic |
| 19 | IC445597 | South | AP-1 | Tella hamsa | HR-12 x T(N)1 | DT | Tolerant to cold in vegetative state, tolerant to blast, superfine grain, Grains: LS, Cold tolerant, moderately susceptible to blast and moderately resistant to SB. |
| 20 | IC469281 | South | AP-31 | NLR-33057 | IR-36 x MTU-4569 (IET-11582) | DT | Dwarf (80-85 cm), grains: LS, white, AWA, resistant to blast, tolerant to *Helminthosporium* & BLB, susceptible to RTV, SB, LF, RH, rice thrips & BPH; Yield: 60 Qxha. |
| 21 | IC469282 | South | AP-32 | NLR-145 | CICA-4 x IR-625-23-3-1 x Tetep (Swarnamukhi) | DT | Semi dwarf (80-85 cm), grains: LS white, AWA, highly resistant to blast, moderately resistant to BS, tolerant to SB, GM, susceptible to BB, BPH & RL; Yield: 65-70 Qxha. |
| 22 | IC469283 | South | AP-33 | NLR-33358 | Selection from IR-50 Somasila (NLR-33358)(IET-13932) | DT | Dwarf (80-85 cm), grains: MS, white, AWA, resistant to blast and *Helminthosporium*, tolerant to BLB, susceptible to RTV; Yield: 60 Qxha. |
| 23 | IC469284 | South | AP-34 | NLR-30491 | IR-36 x IR-2508 (Bharani (NLR-30491)(IET-12630)) | DT | Dwarf (70-75 cm), grains: LS, white, AWA, resistant to *Helminthosporium* & RTV, tolerant to GF & SB, susceptible to blast, LF, RH & BPH; Yield: 60 Qxha. |
| 24 | IC469285 | South | AP-35 | NLR-33359 | Selection from IR-50 (Sravani (NLR-33359)(IET-14876)) | DT | Dwarf (80-85 cm), grains: LS, white, resistant to blast, *Helminthosporium*, tolerant to BLB, susceptible to RTV; Yield: 60 Qxha |
| 25 | IC443796 | South | AP-25 | Early Samba (RNRM-7) | Mutant of BPT-5204 | RV/DT | Dwarf variety, medium slender,white grain, tolerant to SB |
| 26 | IC140227 | South | AP-20 | Nandi (MTU-5182) | Sowbhagya x ARC-6650 | RV/DT |  |
| 27 | IC443794 | South | AP-22 | Vijetha (MTU 7014) | MTU-5249 x MTU-7014 | RV/DT | high yielding |
| 28 | IC443795 | South | AP-23 | Cottondora Sannalu (MTU-1010) | Krishnaveni x IR-64 | RV/DT | Resistant to Blast |
| 29 | IC302937 | South | AP-24 | Deepti (MTU-4870) | Sowbhagya x ARC-6650 | RV/DT | High yielding, non lodging , posses good cooking quality, recommended for saline soil |
| 30 | IC305092 | South | AP-6 | Prasanna | RP-1667-301-1196-1562 (IET-7564) IRAT-8 x N-22 | RV/DT | Fine grained , suitable for rain fed areas |
| 31 | IC443793 | South | AP | Raja Vadlu (RNR-99377) | Rajendra x IR-30 | RV/DT | Superfine grain, tolerant to Sh.R also |
| 32 | IC397687 | South | AP | JGL-1798 (Jagtyal sannalu) | Sambha Mahsuri x Kavya | RV | Superfine grain, resistant to GM, tolerant to blast and BLB |
| 33 | IC75534 | South | KA-19 | Prakash | T-90 x IR-8 (Prakash (IET-2254) | DT | Dwarf (85-88 cm), grains: LS, moderately resistant to blast, BLB, GLH, SB and moderately susceptible to RTV, Yield: 64 Qxha. |
| 34 | IC296242 | South | KA-2 | IET 7575(Sonasali) | RP-1015-348-85-1 x Sona x Manoharsali | DT |  |
| 35 | IC296247 | South | KA-6 | Vibhava | CR-44-35 x W-12708 | DT | Semi dwarf, grains: super fine, white, resistant to GM, moderately resistant to SB, blast & Sh. B.; Yield: 55 Qxha. |
| 36 | IC443851 | South | KA-9 | Sharavathi | (FRR843-3/1R 3874-137-2-55) x (FRR843/1R 38787-26-2-2-3) | DT | Bold grain, red rice, recommended for hilly region |
| 37 | IC443853 | South | KA-12 | KMR 3R |  | DT |  |
| 38 | IC443854 | South | KA-13 | Bili Mukhti (CTH-3) | Selection from IR 9202-251-3 | DT | Long , bold |
| 39 | IC443855 | South | KA-14 | KHP-2 | IR-10781 | DT | Grains: long slender, white, tolerant to leaf blight |
| 40 | IC443856 | South | KA-15 | IET 13901 (Tunga) | Progeny of (Pankaj x Mahsuri) x TKM 6 | DT | long slender grain, tolerant to blast and BLB |
| 41 | IC443858 | South | KA-17 | IET 7191 | RP-5-32 x Pankaj | DT | Dwarf (80-85 cm), grains: MS, Yield: 40-50 Q/ha. |
| 42 | IC443852 | South | KA-11 | Mugad sugandha – 1 | Selection from a Basmati composite (RP-ST-328) | RV/DT | Dwarf (65 cm), grains – long slender , moderate resistant to blast, moderate tolerant to LR |
| 43 | IC443850 | South | KA-8 | Mukthi (CTH-1) | SI Renah Merah x IR-2153 | RV/DT | Semi dwarf, Grains: MB, tolerant to blast & cold temperatures; Yield: 40-45 Qxha. |
| 44 | IC443848 | South | KA-4 | IR 30864 | IR-17-38xIR-7801-1-2-1xIR-46 Khaola | DT | Semi dwarf (80-85 cm), grains: LS, tolerant to LB, neck blast, SB & tolerant to BPH; Yield: 40-60 Qxha. |
| 45 | IC443847 | South | KA-3 | IET 8116 | Vikram x Andrewsali | RV/DT |  |
| 46 | IC302926 | South | KA-10 | Hemavathi | Introduction from Bangladesh | RV/DT | Tolerant to Leaf and neck blast, higher yield under temporary submergence conditions and has better cooking quality |
| 47 | IC302927 | South | KA-5 | Karnataka Hill Paddy-5 (KHP-5) | Intan x IET-7191 | RV/DT | Dwarf (85-90 cm), grains: MB, resistant to blast; Yield: 35-40 Qxha. |
| 48 | IC443857**^$^** | South | KA-16 | Karnataka Rice Hybrid-2 (KRH-2) Hybrid -4xIET 15065xKRH-2 | IR-58025 A x KMR-3 R | RV/DT | Grains: long slender, white, tolerant to leaf blight |
| 49 | IC443846 | South | KA-1 | BR 2655 | (BR 10 X BR 4)  X (BR7 X  Palghar 84-3)  BR-2655-9-3-1 | RV/DT | Medium slender grain, blast resistant variety |
| 50 | IC443859 | South | KE-1 | Bhadra (MO-4) | IR 8 x PTB 20 (HS) | DT | Dwarf (81 cm), red, grains: MB, Yield: 55 Qxha. |
| 51 | IC443860 | South | KE-2 | Asha (MO-5) | IR 11 x Kochuvithu (HS) | DT |  |
| 52 | IC443861 | South | KE-3 | Pavizham (MO-6) | IR-8 x Carivennpel | DT | Semi dwarf, grains: short bold, red |
| 53 | IC443862 | South | KE-4 | Karthika (MO-7) | Triveni x IR 15399 (HS) | DT |  |
| 54 | IC443863 | South | KE-5 | Aruna (MO-8) | Jaya x PTB 33 (HS) | DT |  |
| 55 | IC443864 | South | KE-6 | Makom (MO-9) | ARC 6650 x Jaya (HS) | DT |  |
| 56 | IC443865 | South | KE-7 | Remya (MO-10) | Jaya x PTB 33 (HS) | DT |  |
| 57 | IC443866 | South | KE-8 | Kanakam (MO-11) | IR 1561 x PTB 33 (HS) | DT |  |
| 58 | IC443867 | South | KE-9 | Renjini (MO-12) | MO 5 x Improved Sona (Pedigree selection) | DT |  |
| 59 | IC443869 | South | KE-11 | Panchami (MO-14) | Pothana x MO5 (Pedigree selection) | DT | Grains: MB, tolerant to GM. |
| 60 | IC443875a | South | KE-17 | Gauri | Rajeshwari x Vikram (IET-7428) | DT | Dwarf (80cm), grains: short slender, moderately drought tolerant; Yield: 25 Qxha. |
| 61 | IC443876 | South | KE-18 | Swetha | IR 50 x C 148 (a land race from Andaman) | DT | Semi tall, non lodging, medium tillering and photoperiod insensitive variety, moderate resistance to GM and SB |
| 62 | IC469299 | South | KE-19 | PTB-1 (Aryan) | Pureline selection from Aryan | DT | Long bold grain, moderately resistant to blast |
| 63 | IC469300 | South | KE-20 | PTB-2 | Pureline selection from Ponnaryan | DT | Long bold grain, Good milling and cooking qualities |
| 64 | IC469301 | South | KE-21 | PTB-5 | Pureline selection from Velutharikayama | DT | Long bold grain, moderately resistant to major pests and diseases |
| 65 | IC469302 | South | KE-22 | PTB-7 | Pureline selection from Parambuvattan | DT | Medium bold grain, resistant to foot rot and GM, SB, withstands drought |
| 66 | IC469303 | South | KE-23 | PTB-8 | Pureline selection from Chuvannari Thavalakannan | DT | Medium bold grain, GLH resistant, good milling and cooking qualities |
| 67 | IC469304 | South | KE-24 | PTB-9 | Pureline selection from Veluthari Thavalakannan | RV/DT | Long bold grain, moderately resistant to major pests and diseases, good milling and cooking qualities |
| 68 | IC469305 | South | KE-25 | PTB-10 | Pureline selection from Veluthari Thekkancheera | DT | Photoinsensitive variety, shows good combining ability, resistant to BPH, GM and SB |
| 69 | IC469306 | South | KE-26 | PTB-13 | Pureline selection from Kayama | DT | Aromatic, short bold grain |
| 70 | IC469307 | South | KE-27 | PTB-14 | Pureline selection from Maskathi | DT |  |
| 71 | IC469308 | South | KE-28 | PTB-17 | Pureline selection from Jedduhalliga | DT | Short bold grain, moderately resistant to blast |
| 72 | IC469309 | South | KE-29 | PTB-22 | Pureline selection from Veluthavattan | DT | Bacterial Blight resistant, moderately resistant to blast |
| 73 | IC469310 | South | KE-30 | PTB-23 | Pureline selection from Cheriya Aryan | DT | Good milling and cooking qualities, moderately resistant to blast |
| 74 | IC469311 | South | KE-31 | PTB-24 | Pureline selection from Chuvannavattan | DT | Long bold grain, moderately resistant to blast |
| 75 | IC469312 | South | KE-32 | PTB-25 | Pureline selection from Thonnooran | DT | Moderately resistant to blast |
| 76 | IC469313 | South | KE-33 | PTB-26 | Pureline selection from Chenkayama | DT | Moderately resistant to blast |
| 77 | IC469314 | South | KE-34 | PTB-28 | Pureline selection from Kattamodan | DT | Good milling and cooking qualities, moderately resistant to blast and drought |
| 78 | IC469315 | South | KE-35 | PTB-29 | Pureline selection from Karuthamodan | DT | Moderately resistant to blast, bacterial blight and drought |
| 79 | IC469316 | South | KE-36 | PTB-31 | Pureline selection from Elappapoochampan | DT |  |
| 80 | IC469317 | South | KE-37 | PTB-35 (Annapoorna) | PTB20 x IR-8 | DT | Short, bold and red grain, susceptible to blast, sheath blight and brown plant hopper |
| 81 | IC469318 | South | KE-38 | PTB-36 (Rohini) | PTB 10 x IR-8 | DT | Grains: long bold, white, resistant to blast, Yield: 20 Qxha. |
| 82 | IC469319 | South | KE-39 | PTB-37(Aswathi) | PTB 10 x Dee-gee-woo-gen | DT | Grains: long bold, white, moderately resistant to GM, moderately susceptible to BLB and BPH, Yield: 50 Qxha. |
| 83 | IC469320 | South | KE-40 | PTB-38 (Triveni) | Annapoorna x PTB-15 | DT | Dwarf, grains: short bold, AWP, white, moderately resistant to blast, Yield: 60 Qxha. |
| 84 | IC469321 | South | KE-41 | PTB-39 | PTB 10 x IR-8 (HS) | DT |  |
| 85 | IC469322 | South | KE-42 | PTB-40 | IR8x2 x Annapoorna (HS) | DT |  |
| 86 | IC469323 | South | KE-43 | PTB-41 | PTB 10 x IR-8 (HS) | DT |  |
| 87 | IC469324 | South | KE-44 | PTB-43 | Bhavani x Triveni (HS) | DT |  |
| 88 | IC469325 | South | KE-45 | PTB-45 | Reselection from Triveni | DT |  |
| 89 | IC469326 | South | KE-46 | PTB-46 | IR 2061 x Triveni (HS) | DT |  |
| 90 | IC469327 | South | KE-47 | PTB-47 | IR 20 x IR 5 (HS) | DT |  |
| 91 | IC469332 | South | KE-52 | PTB-53 | Reselection from Mashuri | DT |  |
| 92 | IC469334 | South | KE-54 | Harsha (PTB-55) | M210 x PTB28 | DT | Long, bold and red grain, moderately resistant to blast and moisture stress |
| 93 | IC469329 | South | KE-49 | Kanchana (PTB-50) | BR 51 - 46 -1 x Cul 23331 - 1 (HS) or IR-36xPavizham | RV/DT | Tall (88 cm), long and bold grains, mod. resit. to leaf blast, Sh. B., brown spot, RTV & SB |
| 94 | IC469330 | South | KE-50 | Aathira (PTB-51) | Jyothi x BR-51-46-1 | RV/DT | Tall (110 cm), grains: Short Bold, mod. resit. to leaf blast, neck blast, Sh. B. and tolerant to GM, BPH & WBPH |
| 95 | IC469331 | South | KE-51 | Aiswarya (PTB 52) | Jyothi x BR-51-46-1 | RV/DT | Tall (108 cm), grains : Long Bold, mod. resit. to leaf blast, neck blast, Sh. B. and tolerant to GM-1 & 2, BPH |
| 96 | IC469328 | South | KE-48 | Kairaly (PTB 49) | IR 36 x Jyothi (HS) | RV/DT | Tall (90 cm), grains : Long Bold, multiple resit. to leaf blast, neck blast, GM biotypes GM-1 & GM-4, WBPH and GM-1 |
| 97 | IC422088 | South | KE | Deepthi (WND III) | Pure line Selection from Edavaka | RV | Tall (123 cm), grain: LS, red, medium duration, resistant to *Udbatta* and moderately resistant to blast, LR and SB; Yield: 40 Qxh |
| 98 | IC469333 | South | KE-53 | Karuna (PTB 54) | Bhavani x Triveni | RV/DT | Tall (147 cm), grains: LB, red, moderately resistant to Sh.B and blast, SB; Yield: 45-50 Qxha. |
| 99 | IC443868 | South | KE-10 | KAU M 59-29-2-1-2 (GM1) (MO 13 - Pavithra) | Surekha x MO-5 | RV/DT | Grains: MB, tolerant to GM. |
| 100 | IC443874 | South | KE-16 | Krishna Anjana (MO-19) KAU M 57-187-1-1 (K 18) | MO-1 x MO-6 | RV/DT | Grains: MB, resistant to BPH and iron toxicity. |
| 101 | IC443871 | South | KE-13 | UMA (MO-16) KAU M 42-6-3 (D1) | MO-6 x Pokkali | RV/DT | Grains: MB, tolerant to GM, resistant to GM and BPH |
| 102 | IC443872 | South | KE-14 | KAU M 45-20-1 (D6) (MO 17-Revathy) | Cul. 12814 x MO-6 | RV/DT | Grains: MB, tolerant to BPH, resistant to BPH. |
| 103 | IC443873 | South | KE-15 | KAU M 57-9-1-1- (K16) (MO 18-Karishma) | MO-I x MO-6 | RV/DT | Grains: MB, resistant to BPH, iron toxicity and GM |
| 104 | IC443870 | South | KE-12 | KAUM 20-19-4 (MO 15-Remanika) | Mutant to MO-1 | RV/DT | Grains: short bold, resistant to BPH and GM |
| 105 | IC393780 | South | KE | Dhanya (Kym 4) | Jaya x PTB (HS) | RV | Tall (165 cm), mod. resit. to blast, Sh.B, blast SB and LF |
| 106 | IC393781 | South | KE | Sagara (Kym 5) | Orumundakan local (MS) | RV | Tall (165 cm), Tolerant to blast, Sh.B, SB and susceptible to LF |
| 107 | IC296060**^A^** | South | KE | Vytilla 4 | Chettivirippu x IR 4630-22-2-17 (HS) | RV | Salt tolerant variety |
| 108 | IC296061**^A^** | South | KE | Vytilla 5 | Mashuri (Mutant) | RV |  |
| 109 | IC422081 | South | KE | KAU M 61-6-1-1-2 (GM 9) (MO 14-Panchami) IET 14260 | Pothana x MO-5 | RV | Grains - medium bold , tolerant to GM |
| 110 | IC393784 | South | KE | Chingam (Kym 8) | PTB 20 mutant | RV | Semi tall (147 cm), tolerant to Short Bold, mod. tolerant to Sh.B & LS |
| 111 | IC393785 | South | KE | Dhanu (Kym 9) | PTB 9xxIR 8xPTB 28 mutant | RV | Semi tall (123 cm), grains : Short Bold, mod. tolerant to blast, Sh. B, BS & SB |
| 112 | IC113129**^A^** | South | TN-16 | CO 43 | Dasal x IR-20 | DT | Tolerant to saline and alkaline soils, moderately resistant to BS, LB, tolerance to GLH |
| 113 | IC113130 | South | TN-17 | CO 45 | R.Heenati x IR-340-3-267-1 | DT | Dwarf (75-80 cm), grains: LS, AWA, resistant to GLH, SB, blast, BLB, RTV & moderately resistant to Sh.R; Yield: 57 Qxha |
| 114 | IC113137**^A^** | South | TN-15 | Bhavani (CO - 63) | Peta x BPI-76 | DT | Semi tall, grains: long bold, AWP, moderately resistant to blast, BLB, moderately susceptible to BPH |
| 115 | IC443913**^A^** | South | TN-1 | ADT 36 | Triveni x IR-20 | DT | Resistant to BPH and blast |
| 116 | IC443914**^A^** | South | TN-2 | ADT 37 | BG-280-1-2 x PTB-33 | DT | Resistant to many pests and diseases, seed dormancy for 60 days, Semi dwarf (95 cm ), grains: short bold, resistant to leaf yellowing BS, blast, BPH, GLH, moderately resistant to BLB, RTV, GM & LH; Yield: 63 Qxha. |
| 117 | IC443915 | South | TN-3 | ADT 38 | IR-1529-680-3-2 x IR-443252-6-4 | DT | Resistant to BPH, GLH, leaf folder, susceptible to BLB, Dwarf (80-85 cm), grains: MS, white, resistant to blast, BS, GLH, WBPH, moderately resistant to RTV, BPH & GM; Yield: 58 Qxha. |
| 118 | IC443916 | South | TN-4 | ADT 39 | IR-8 x IR-20 | DT | Dwarf (76 cm), grains: MS, white, AWA, resistant to blast, BB & Sh.R; Yield: 56 Qxha. |
| 119 | IC443917 | South | TN-5 | ADT 40 | RPW 6-13 x Sona 2 | DT | Suitable to water logged situation, short bold white rice |
| 120 | IC443919**^A^** | South | TN-7 | ADT 42 | AD 9246 x ADT 29 | DT | Moderately resistant to blast and BPH |
| 121 | IC443922**^A^** | South | TN-12 | ASD 16 | ADT-31 x CO-39 | DT | Tolerant to salinity, resistant to blast, medium tillering, more number of grains per panicle. |
| 122 | IC443925**^A^** | South | TN-20 | I. W. Ponni | Selection from white Ponni | DT | Tall (130-135 cm), grains: fine, white, AWA, resistant to RTV, moderately resistance to brown spot & blast; Yield: 45 Qxha. |
| 123 | IC443926 | South | TN-21 | Ponmani | Pankaj x Jagannath | DT | Resistant to Brown plant hopper and high yield potential. |
| 124 | IC469392 | South | TN-22 | ASD-1 | Pureline | DT | Red rice variety |
| 125 | IC469393 | South | TN-23 | ASD-2 | Pureline | DT | Non shedding |
| 126 | IC469394 | South | TN-24 | ASD-3 | Pureline | DT | Non lodging and drought resistant variety |
| 127 | IC469395 | South | TN-25 | ASD-7 | Pureline | DT | High yielder |
| 128 | IC469396 | South | TN-26 | ASD-9 | Pureline | DT | Red rice variety |
| 129 | IC469397 | South | TN-27 | ASD-15 | IR 26 x IR 22 | DT | High yielding fine rice |
| 130 | IC469398**^A^** | South | TN-28 | ASD-17 | ADT-31 x Ratna x ASD-8 x IR-8 | DT | Semi dwarf (96 cm), grains: short bold, red, moderately susceptible to GM, WBPH, moderately resistant to BPH; Yield: 54 Qxha. |
| 131 | IC302978 | South | TN-18 | CO-46 | T 7 x LR 20 | RV/DT | Resistant to BPH |
| 132 | IC443923**^A^** | South | TN-13 | ASD-18 | ADT-31 x IR-50 | RV/DT | Resistant to blast, Semi dwarf (90 cm), grains: slender, white, resistant to blast, moderately resistant to Sh.R, RTV, resistant to WBPH, BPH and moderately resistant to GM; Yield: 51 Q.xha. |
| 133 | IC469399**^A^** | South | TN-29 | ASD-19 | Lalnakanda x IR-30 | RV/DT | Drought tolerant |
| 134 | IC443924**^A^** | South | TN-14 | ASD-20 | IR-18348-38-3 x IR-25863-61-3-2 x IR-58 | RV/DT | Resistant to many pests and diseases |
| 135 | IC302979**^A^** | South | TN-19 | CO 47 | IR-50 x CO-43 | RV/DT | Dwarf (80-92 cm), grains: MS, resistant to blast, moderately susceptible to all hoppers |
| 136 | IC443918 | South | TN-6 | ADT-41 | Selection from a dwarf mutant of Basmati-370 | RV/DT | Semi dwarf (95-105 cm), grains: extra LS, mild aroma susceptible to major pest & disease of rice; Yield: 47 Qxha. |
| 137 | IC302924**^A^** | South | TN-8 | ADT-43 | IR-50 x Imported white Ponni | RV/DT | Resistant to GLH, high tillering, moderately resistant to BPH, SB & GM |
| 138 | IC302925 | South | TN-9 | ADT 44 | Selection from OR 128-7-S1 (IET 14099-1256(OR 142-99) | RV/DT | Resistant to blast and GLH |
| 139 | IC443920**^A^** | South | TN-10 | ADT (R) 45 | IR 50 x ADT 37 | RV/DT | Resistant to rice gall midge, medium resistant to SB and BPH |
| 140 | IC443921 | South | TN-11 | ADT (R) 46 | ADT 38 x CO 45 | RV/DT | High yielding variety |
| 141 | IC443901 | South | PY-1 | Paduvai Ponni-1 | Ponni x IR-8 | DT | Grains: MS, tolerant to RTV, Yield: 57 Qxha. |
| 142 | IC443902 | South | PY-2 | Punithavathy | Kannagi x Culture 2032 | DT | Dwarf, grains: MS, white, AWA, tolerant to BPH & RTV; Yield: 47 Qxha. |
| 143 | IC443903 | South | PY-3 | Bharathidasan | IR3408-267 x PTB3311x IR26 | DT |  |
| 144 | IC443906 | South | PY-6 | Subramaniya Bharathi | IR-19661 x CR-1009 | DT | Tall (120 cm), grains: MS, white, AWA, resistant to blast & GM; Yield: 55 Qxha. |
| 145 | IC443904 | South | PY-4 | Jawahar | IR-8 x H-4 | RV/DT | Grains: white, tolerant to BLB, BS, RTV, Sh.R, leaf roller & SB; Yield: 62 Qxha. |
| 146 | IC443905 | South | PY-5 | Aravinder | SwarndhanxNLR-9674 is a selection from IET 10158 | RV/DT |  |
| 147 | IC443805 | North east | AS-7 | Moniram | Pankaj x Mahsuri | DT | Leaf blast tolerant, Sheath Blight tolerant |
| 148 | IC443806 | North east | AS-8 | Piolee | Pankaj x Mahsuri | DT | Blast resistant and moderately resistant to bacterial leaf blight |
| 149 | IC443808**^A^** | North east | AS-10 | Kapilee | Heera x Annada | DT | Semi dwarf variety, long and bold grain, resistant to bacterial leaf blight, moderately resistant to stem borer and gall midge |
| 150 | IC443809 | North east | AS-11 | Basundhara | IET-9711 x IET-11161 | DT | Resistant to GM, BPH, BLB and moderately resistant to blast, Semi-dwarf (107 cm), grains: MS, white, resistant to blast, BLB, GM, BPH, SB, LF; susceptible to Sh.B.; Yield: 40 Qxha. |
| 151 | IC443810 | North east | AS-12 | Jayamati | Jaya x Mahsuri (IET-13253) | DT | Tall (130 cm), grains: MS, white, suitable for Boro, resistant to blast, BLB, SB & GM, susceptible to Sh.B., Yield: 65 Qxha. |
| 152 | IC469286**^A^** | North east | AS-17 | Manoharsali | Lati sali x Guachari | DT | Tall (138 cm), grains: long bold, moderately resistant to blast, moderately susceptible to BLB and resistant to GM. |
| 153 | IC469287 | North east | AS-18 | KMJ-1-172 | IR8 x Manoharsali | DT |  |
| 154 | IC469288 | North east | AS-19 | KMJ-1-192 | IR8 x Manoharsali | DT |  |
| 155 | IC469290 | North east | AS-21 | Pamindra | Pankaj x Nagoba | DT | Medium bold grains, resistant to blast. |
| 156 | IC443799**^A^** | North east | AS-1 | Chilarai | IR-24xCR 44-118-1 | RV/DT | Semi dwarf (95 cm), grains: white, AWA, resistant to grassy stunt virus, moderately resistant to BLB & Sh.B; Yield: 30 Qxha. |
| 157 | IC443800**^A^** | North east | AS-2 | Lachit | CRM 13-3241xKalinga II | RV/DT | Semi dwarf (95 cm), grains: white, AWP; Yield: 30 Qxha. |
| 158 | IC443803 | North east | AS-5 | Bahadur | Pankaj x Mahsuri | RV/DT | Semi dwarf (114 cm) plant with compact panicle with brown husk colour, AWP, resistant to LB, tolerant to neck blast, BLB, moderately susceptible to Sh.B, SB & susceptible to GM; Yield: 40 Qxha. |
| 159 | IC443801 | North east | AS-3 | Bhogali | Ghewbora x KMJ 1-52-2 | RV/DT | Tall (145 cm), tolerant to glume blight, susceptible to BS & GM; Yield-36 Qxha. |
| 160 | IC443804 | North east | AS-6 | Kushol | Pankaj x Mahsuri | RV/DT | Semi dwarf (116 cm), grains: fine, white, resistant to LB, neck blast, tolerant to BLB & moderately susceptible to GM; Yield - 40 Qxha. |
| 161 | IC443802 | North east | AS-4 | Rongilee | Ghewbora x KMJ-1-52-2 | RV/DT | Tall (150 cm), tolerant to glume blight & BLB, susceptible to BS & GM; Yield-35 Qxha. |
| 162 | IC443812**^A^** | North east | AS-14 | Luit | Heera x Annada (Designated as TTB 127-216-2) | RV/DT | Grains: medium bold, resistant to blast, Yield: 35 Qxha. |
| 163 | IC443807 | North east | AS-9 | Ranjit | Pankaj x Mahsuri | RV/DT | Semi dwarf (99 cm) with quality grain of short fine, tolerant to BLB susceptible to blast, SB & GM; Yield: 40 Qxha. |
| 164 | IC443814 | North east | AS-16 | Aghoni | Gandhi bora x KMJ-1-52-2 | RV/DT | Parboiled soak and eat rice variety, medium bold grain |
| 165 | IC443811 | North east | AS-13 | Ketekijoha | Savithri x Bhadshabhog | RV/DT | Grains: MS, resistant to blast |
| 166 | IC469289 | North east | AS-20 | Padmanath | Pankaj x Jagannath x Nagoba | RV/DT | Grains: Long Bold |
| 167 | IC443813 | North east | AS-15 | Satyaranjan | IET-9711 x IET-11162 | RV/DT | Tall (113 cm), grains: MS, white, resistant to blast, LF, GM, WBPH, BPH, susceptible to BLB & Sh.B; Yield: 40 Qxha. |
| 168 | IC469344 | North east | ME-5 | Shah Sharang-1 | Mirikrack x Rasi | DT | Tolerant to iron toxicity, suitable for lowland in mid altitude areas |
| 169 | IC469345 | North east | ME-6 | Khonorullo | Local variety | DT | Grains short bold , recommended for rain fed lowland ecosystems of high altitude areas, tolerant to sheath rot and low temperature at reproductive phase |
| 170 | IC469346 | North east | ME-7 | Lum Pnah-1 | IR-29 x Naoba | DT | Glutinous variety, tolerant to blast, suitable for lowland in mid altitude areas |
| 171 | IC469347 | North east | ME-8 | Ngoba | Local collection from Meghalaya | DT | Glutinous, non aromatic, moderately resistant to SB |
| 172 | IC469340 | North east | ME-1 | Bha Lum-1 | PSN x Line N. 6131. | RV/DT | Intermediate (105-115 cm); grains - long bold, highly resist. to blast, mod. resist. to SB |
| 173 | IC469341 | North east | ME-2 | Bha Lum-2 | Cross PSN x Line No. 6131. | RV/DT | Intermediate (100-110 cm); grains - long bold, highly resist. to blast, mod. resist. to SB |
| 174 | IC469342 | North east | ME-3 | NEH Megha Rice 1 | Pusa 33 x Khonorullu | RV/DT | Non Aromatic , suitable for rain fed lowland ecosystem of high altitude areas, tolerant to low temperature at reproductive phase |
| 175 | IC469343 | North east | ME-4 | NEH Megha Rice 2 | KhonorolluxPusa 33 | RV/DT | Suitable for late transplanting |
| 176 | IC443896 | North east | MNP-1 | RCM-5 (RcManiphou-6) | CH988 x IR24 | DT | Tall (120-130 cm), long medium grain, moderately resistant to leaf & neck blast, Sh.B & SB |
| 177 | IC443897 | North east | MNP-2 | RCM-9 (Rc Maniphou-7) | Mutant culture from Punshi | DT | Semi dwarf (100 cm), long medium grain, moderately resistant to leaf blast, Sh.B & SB |
| 178 | IC443816 | East | BI-2 | Janki | Pure line selection from Chenab Rice | DT | Deep water rice, resistant to tungro, Tall, lodging tendency, grains: long bold, moderately resistant to RTV & BPH, Yield: 15-20 Qxha |
| 179 | IC443817 | East | BI-3 | Kanak | Jaya x BR-34 | DT | Resistant to BPH, Semi dwarf (100–110 cm), grains: long bold, resistant to BB, moderately resistant to SB & LH; Yield: 40-45 Qxha. |
| 180 | IC443818 | East | BI-4 | Radha | IR-20 x IR-5-114-3 (BR-51-91-6) | DT | Resistant to BLB moderately resistant to Sh.R, RTV, SB & GLH |
| 181 | IC443820 | East | BI-6 | Sugandha | Pure line selection from Basmati Rice | DT | Tall (130-140 cm), grains: MS with aroma, white, moderately resistant to BLB and Pest complex. |
| 182 | IC443821 | East | BI-8 | Sneha | Annanda x CR-143-2-2 | DT | Semi dwarf (80-85 cm), grains: short bold, resistant to RTV, blast, susceptible to major diseases & pests; Yield: 35 Qxha. |
| 183 | IC443822 | East | BI-9 | Jaishree | Jaya x Mahsuri | DT | Resistant to BLB, Tall (120-135 cm), grains: MS, white, high head recovery, moderately resistant to blast, BLB, SB & GLH, Yield: 35-40 Qxha. |
| 184 | IC248491 | East | BI-13 | Rajshree | Pure line selection | DT | Tall (130-135 cm), grains: MS, white, resistant to BB, BS, Sh.R & moderately resistant to SB; Yield: 35-40 Qxha. |
| 185 | IC469291 | East | BI-10 | Santosh | Pankaj x BR34 | DT | Suitable for rain fed areas, multiple disease resistance, long slender grain. |
| 186 | IC469292 | East | BI-11 |  |  | DT |  |
| 187 | IC469293 | East | BI-12 | Kishori | IR-8 x Barogar | DT | Low land |
| 188 | IC469294 | East | BI-14 | Richari | IET-7564 x Pusa 33 | DT |  |
| 189 | IC469295 | East | BI-15 | Satyam | RD-19 x Desaria | DT | BPH resistant, medium-long grain |
| 190 | IC469296 | East | BI-16 | Sudha | Pure line selection | DT | Semi dwarf (110-120 cm), grains: long bold, red, resistant to RTV, BS, Sh.R, SB, moderately resistant to BB; Yield: 20-25 Qxha |
| 191 | IC469297 | East | BI-17 | Vaidehi | Pure line selection from Beldar (TCA-48) | DT | Deep water rice, Tall, grains: long bold, resistant to blast, BLB, tolerant to drought; Yield: 30-35 Qxha. |
| 192 | IC443819 | East | BI-5 | Shakuntala | Pankaj x BR-8 (RAU 73-16-1-40) | RV/DT | Low land |
| 193 | IC305003**^A^** | East | BI-7 | Vandana | C22 x Kalakeri | RV/DT | Tall (95-155 cm), long bold, white grain, moderately resistant to termite & SB , blast & BS |
| 194 | IC443815 | East | BI-1 | Gautam | Rasi Mutant | RV/DT | Dwarf( 70 cm), long bold, white grain |
| 195 | IC443878 | East | MP-2 | Kranti | Cross-16 x IR-8 (R-2022) | DT | Semi dwarf (130 cm), grains: short bold, AWP, Yield: 50 Qxha, low land rice |
| 196 | IC443882 | East | MP-6 | Dubraj | Traditional cultivar of MP | DT |  |
| 197 | IC443881 | East | MP-5 | Poornima | Poorva x IR-8608-298 | RV/DT | Semi-dwarf (90-100 cm), grains: LS, white, resistant to major diseases & pests; Yield: 30 Qxha |
| 198 | IC443879 | East | MP-3 | Shyamala | R-60-2713 x R-2386 | RV/DT | Semi-dwarf (80-90 cm), grains: LS, moderately resistant to leaf blight & GM; Yield: 28 Qxha |
| 199 | IC443880 | East | MP-4 | Bamaleshwari | RP 2151-40-1 x IR 9828-23 | RV/DT |  |
| 200 | IC443877 | East | MP-1 | Danteswari | Samridhi x IR 8608298 | RV/DT |  |
| 201 | IC443875b | East | OR-38 | Gauri | Rajeshwari x Vikram | DT | Dwarf (80cm), grains: short slender, moderately drought tolerant; Yield: 25 Qxha. |
| 202 | IC443898**^A^** | East | OR-1 | Annada | MTU-15 x Yaikaku Nantoku (China) (IET-6223) | DT | Grains: short bold, moderately resistant to blast & SB, susceptible to BLB, GM & BPH. |
| 203 | IC443899 | East | OR-2 | Gayatri | Pankaj x Jagannath (IET-8020) | DT | Semi dwarf (100 cm), grains: short bold, resistant to BLB, moderately resistant to blast & GM; Yield: 40-60 Qxha. |
| 204 | IC443900 | East | OR-3 | Utkal Prava | Waikoku x CR-1014 | DT | Tall, grains: MS, susceptible to BS, Yield: 35-40 Qxha. |
| 205 | IC469348 | East | OR-4 | Rajeswari | T-90 x IR-8 | DT | Dwarf (80 cm), grains: short bold, moderately resistant to blast,  BLB & GLH, susceptible to SB & GM. |
| 206 | IC469349 | East | OR-5 | Pathara | Hema x CO 18 | DT |  |
| 207 | IC469350 | East | OR-6 | Subhadra | T(N)1 x SR26B | DT |  |
| 208 | IC469352 | East | OR-8 | Shankar | Parijat x IET-3225 | DT |  |
| 209 | IC469354**^A^** | East | OR-10 | Pariijat | TKM-6 x T(N)1 | DT | Dwarf (85 cm), grains: MS, moderately resistant to SB, GLH, Blast, BLB & *Helminthosporium*, Yield: 30-40 Qxha. |
| 210 | IC469355 | East | OR-11 | Pratap | Kumar x CR-57-49 (OR-131-3-1) | DT | Dwarf (73 cm), moderately resistant to blast and SB, resistant to GM, BPH, GLH & *Helminthosporium* |
| 211 | IC469356 | East | OR-12 | Daya | Kumar x CR-57-49 (OR-131-13-13) | DT | Dwarf (70 cm), moderately resistant to blast, SB and BLB, resistant to GM, BPH, GH & *Helminthosporium*, Yield: 40 Qxha |
| 212 | IC469358 | East | OR-14 | Keshari | Kumar x Jagannath | DT | Short stature, grains: MS, moderately resistant to GLH, blast and BLB, Yield: 30-40 Qxha. |
| 213 | IC469359 | East | OR-15 | Lalithagiri | Badami x IR-1966-364 | DT | Semi-dwarf, grains: MB, white, resistant to blast and tolerant to BPH; Yield: 25-35 Qxha |
| 214 | IC469360 | East | OR-16 | Hema | CR-5-11 | DT | De notified variety |
| 215 | IC469361 | East | OR-17 | Suphala | T-141 x TN-1 | DT |  |
| 216 | IC469363 | East | OR-19 | Ananga | Kumar (T-90 x IR-8) x CR57-49 (IET-7433) | DT | Dwarf (75 cm), grains: MS, resistant to GM, BPH, moderately resistant to blast, BLB, RTV & drought; Yield: 50 Qxha. |
| 217 | IC469364 | East | OR-20 | Lalat | Obs.677 x IR-207 x Vikram (IET-9947) | DT | Dwarf (85-90 cm), grains: LS, resistant to Sh.R, GM, BPH, GLH, moderately resistant to blast, Sh.B, BS, RTV, BLB & SB; Yield: 40 Qxha. |
| 218 | IC469366 | East | OR-22 | Shrabani | Selection from IR-50 | DT |  |
| 219 | IC469367 | East | OR-23 | Jajati | Rajeshwari x T-141 | DT | Grains: MS, moderately resistant to GLH, blast and *Helminthosporium* |
| 220 | IC469368 | East | OR-24 | Sarthi | T-90 x IR-8 x W-1263 | DT | Dwarf (75-80 cm), resistant to blast, BLS, GM, GLH, moderately resistant to BLB, blast and BPH |
| 221 | IC469369 | East | OR-25 | Rudra | Parjat x IET 3225 | DT |  |
| 222 | IC469378 | East | OR-34 | Indravathi | IR-56 x OR-142-99 | DT | Semi-dwarf, grains: MB, white, Yield: 45-65 Qxha. |
| 223 | IC469380 | East | OR-36 | Sebati | Daya x IR-36 | DT | Semi-dwarf, grains: MS, white, resistant to blast; Yield: 35-50 Qxha. |
| 224 | IC469381 | East | OR-37 | Jagabandhu | Savitri x IR-4819 Sel. x IR-27301 Sel. | DT | Semi dwarf (103 cm), grains: MB, white, moderately resistant to lodging and shattering |
| 225 | IC469382 | East | OR-39 | Rambha | Pankaj x W1263 | DT |  |
| 226 | IC469384 | East | OR-41 | Bhuvan | OR-158(IR-8xSiam-29) x Parijat x Rasi | DT |  |
| 227 | IC469389 | East | OR-46 | Jagannath | Mutant of T-141 (BSS-873) | DT | Semi Dwarf (90-110 cm), grains: MS, resistant to lodging, blast & SB, Yield: 40-50 Qxha. |
| 228 | IC469371 | East | OR-27 | Kanchana | Jajati x Mehsuri | RV/DT | Tall (120-150 cm), grains: MS, resistant to blast, BLB, moderately resistant to Sh. B, BPH, & resistant to GLH; Yield: 40 Qxha. |
| 229 | IC469388 | East | OR-45 | Bhanja | IR-36 x Hema x Vikram (IET-10738) | RV/DT | Semi dwarf (90-95 cm), grains: MB, resistant to blast, Sh.R, GM, moderately resistant to BS & SB; Yield: 40 Qxha. |
| 230 | IC469357 | East | OR-13 | Nilagiri | Suphala x DZ-192 | RV/DT | Grains: MB, resistant to blast, WM, SB., moderately resistant to RTV, neck blast, BLB, Sh.R, BS, RH, GM and LF; Yield: 35 Qxha. |
| 231 | IC469353 | East | OR-9 | Badami | Shuphala x Annapurna | RV/DT | Grains: MB, resistant to LS, GM, LF, moderately resistant to blast, BLB, BPH, SB & good respond to late sown condition; Yield: 35 Qxha. |
| 232 | IC469351 | East | OR-7 | Ghanteswari | IR-2061-628-1-6-4-3 x N-2-2 | RV/DT | Dwarf (85 cm), grains: MB, resistant to blast, neck blast, GM, SB, moderately resistant to Sh.B, BS, BLB, Sh.R & RTV; Yield: 35 Qxha. |
| 233 | IC469365 | East | OR-21 | Khandagiri | Parijat x IR-13429-94-3-2-2 | RV/DT | Grains: MS, white, resistant to Sh.R, neck blast, BS, BPH, moderately resistant to RTV, Sh.B, BLB, WBPH, GM, SB & WM; Yield: 35 Qxha |
| 234 | IC469370 | East | OR-26 | Birupa | ADT 27xIR-8xAnnapurna | RV/DT | Semi dwarf (90-95 cm), grains: MB, white, resistant to blast, BLB, RTV, Sh.B, GM, WM, RH, cutworm & WBPH, Yield: 40 Qxha |
| 235 | IC469387 | East | OR-44 | Manika | OR-210-1010xObs-677 | RV/DT | Grains: MB, resistant to Sh.B, RTV, GM, SB, moderately resistant to blast & BLB; Yield: 45 Qxha. |
| 236 | IC469386 | East | OR-43 | Urbashi | RajeswarixJajati | RV/DT | Grains: MB, resistant GM, moderately resistant to blast, BLB & Sh.R; Yield: 40 Qxha |
| 237 | IC469372 | East | OR-28 | Samanta | T-90xIR-8xVikramxSiam-29xMahsuri | RV/DT | Grains: MB, resistant to GM, SB, moderately resistant to blast, BLB, Sh.R and WBPH; Yield: 45 Qxha. |
| 238 | IC469373 | East | OR-29 | Mehar | OBS-677 x IR-2071 x Vikram x W-1263 | RV/DT | Grains: MB, resistant to GM, BPH, WBPH, WM, neck blast, moderately resistant to SB, Sh.R, BLB, RTV & blast; Yield: 40 Qxha. |
| 239 | IC469377 | East | OR-33 | Gajapathi | OR-136-3 x IR-13429-196-1-120 | RV/DT | Semi-dwarf, grains: MS, white, tolerant to BPH; Yield: 35-50 Qxha. |
| 240 | IC469383 | East | OR-40 | Bhoi | Gauri x RP 825-45-1-3 | RV/DT | Semi-dwarf, grains: MB, white, resistant to blast and BLB; Yield: 35-50 Qxha. |
| 241 | IC469391 | East | OR-48 | Kharaveli | Daya x IR-13240-108-2-2-3 | RV/DT | Semi-dwarf, grains: MS, white, tolerant to BPH; Yield: 35-50 Qxha. |
| 242 | IC469385 | East | OR-42 | Konark | Lalat x OR 135-3-4 | RV/DT | Semi-dwarf, grains: MS, white, tolerant to BPH; Yield: 35-50 Qxha. |
| 243 | IC469390 | East | OR-47 | Mahanadi | OR-1301-13 x IR-19661-131-1-3-1 x Savitri | RV/DT | Semi-dwarf, grains: MB, white, Yield: 45-65 Qxha. |
| 244 | IC469375 | East | OR-31 | Prachi | IR-9764-45-2-2 x OR-149-3-2 | RV/DT | Semi-dwarf, grains: MB, white, photo sensitive, Yield: 45-65 Qxha. |
| 245 | IC469379 | East | OR-35 | Ramachandi | IR-17494-32-2-2-1 x Jagannath | RV/DT | Semi-dwarf, grains: MB, white, photo sensitive, Yield: 45-65 Qxha. |
| 246 | IC469374 | East | OR-30 | Surendra | OR-158-5 x Rasi | RV/DT | Semi-dwarf, grains: MB, white, Yield: 35-50 Qxha. |
| 247 | IC469362 | East | OR-18 | Udayagiri | IRAT-138 x IR-13543-66 | RV/DT | Semi-dwarf, grains: MS, red, Yield: 25-35 Qxha. |
| 248 | IC443936 | East | WB-1 | Dinesh | Jaladhi 2 x Pankaj | DT |  |
| 249 | IC443940 | East | WB-5 | Jogen | IR-26 x SML 40-10-4 (IET-8967) | DT | Tall (110-140 cm), grains: MB, white; Yield: 40 Qxha. |
| 250 | IC443941 | East | WB-6 | Mandira | IR-34 x KLG6987-143-2-9 x IR-270-2-5-6 x HBIDW-8) | DT | Deep water and semi deep water rice |
| 251 | IC443942 | East | WB-7 | Matangini | CN491, a pure line selection from Kayallata | DT | Deep water and semi deep water rice |
| 252 | IC443945 | East | WB-10 | NC-492 | Pure line selection from Boyan | DT |  |
| 253 | IC443939 | East | WB-4 | Jitendra | Selection from land races | RV/DT | Tall (160-170 cm), grains: LS, white, resistant to neck blast, BPH, WBPH, moderately resistant to GM & LF; Yield: 50 Qxha. |
| 254 | IC443943 | East | WB-8 | Neeraja | Selection from land races | RV/DT | Tall (180-200 cm), grains: long bold, yellow, AWA, resistant to BPH & GM, Yield: 35Qxha |
| 255 | IC469400 | East | WB-11 | Bhagirathi | Jhingasali x Patnai-23 | RV/DT | Tall (170-180 cm), grains: short bold, tolerant to Sh.B & SB; Yield: 46 Qxha. |
| 256 | IC443938 | East | WB-3 | Jalaprabha | Selection from composite | RV/DT | Grains: short bold. |
| 257 | IC443944 | North | WB-9 | Saraswathi | Pankaj x Patnai-23 | RV/DT | Grains: long bold. |
| 258 | IC116268 | North | UP-2 | IR 24 | IR 8 x centum Ratna 231 x SLO x sedges | DT |  |
| 259 | IC116270 | North | UP-4 | Manhar | IR-24 x Cauvery (IET-8050) | DT | Dwarf (88 cm), grains: LS, non lodging moderately resistant to BLB, tolerant to WBPH |
| 260 | IC116272 | North | UP-10 | Pantdhan 4 | IR262x Ramadja | DT | Semi Dwarf (115-120 cm), grains: LS, resistant to BLB, LB & BS, moderately resistant to pest complex, Yield: 35-40 Qxha. |
| 261 | IC116273 | North | UP-5 | Pantdhan 6 | IR8608xIR 10179-23 | DT | Tall (113-120 cm), grains: MS; Yield: 41 Qxha. |
| 262 | IC248372 | North | UP-18 | Ashwani | N-22 x Cauvery (KR-5-142) | DT | Dwarf (60-70 cm), grains: short bold, AWP; Yield: 30-31 Qxha |
| 263 | IC248443 | North | UP-11 | Prasad | IR-747-B-26-3 x IR-57948 | DT | Dwarf (90 cm), grains: LS, AWA, highly resistant to BLB, moderately resistant to blast and *Helminthosporium*, resistant to lodging, Yield: 56 Qxha. |
| 264 | IC73566 | North | UP-15 | VL Dhan 16 | JP-5 x YRL-1 | DT | Semi dwarf, grains: red, highly resistant to, SB, leaf and neck blast, Yield: 40-45 Qxha |
| 265 | IC73567 | North | UP-12 | VL Dhan 206 | Pure line selection from local type Bamni | DT | Medium tall, Grains: MS, tolerant to blast and SB, Yield: 20-25 Qxha. |
| 266 | IC73568 | North | UP-16 | VL Dhan 39 | China 1039 x IR580-19-2-3-1 | DT | Semi dwarf (105-110 cm), grains: medium long, white, Yield: 20 Qxha. |
| 267 | IC297620 | North | UP-17 | VL Dhan 163 | IR747kn-16-36-1 x 1R-2053-521-1-1 | DT | Tall, grains:  medium, resistant to drought & low temperature |
| 268 | IC443932 | North | UP-9 | Pantdhan 12 | Govind x UPR-201-1-1 | DT | Medium statured, grains: LS, resistant to BLB, BLS & BPH; Yield: 50-55 Qxha. |
| 269 | IC443934 | North | UP-14 | Pant sugandh dhan 15 | Basmati370 x Sudari x Beharl x Muskan41 | DT | Resistant to gall midge biotype- 1, moderately resistant to stem borer, brown and plant hopper, leaf blast and brown spot |
| 270 | IC443935 | North | UP-19 | Usar 1 | Jaya x Gatu | DT | Dwarf (80-90 cm), grains: short bold, AWP, resistant to BLB & BLS, moderately resistant to insect and pests, Yield: 40-45 Qxha. |
| 271 | IC443928 | North | UP-3 | Jalanidhi | Local Selection from cultivar Goanatis(form dwarf Mutant of Basmati-370) | RV/DT | Tall (180-300 cm), moderately resistant to blast, BS & moderately susceptible to yellow stem borer; Yield: 35-40 Qxha. |
| 272 | IC443930 | North | UP-7 | Jalpriya | Cross IET 4060xJalmagna (IET 4060 x Jalmagna B-B-B-1) | RV/DT | Tall (135-175 cm), grains: LS, moderately resistant to neck blast, BS & yellow steam borer; Yield: 30-35 Qxha. |
| 273 | IC443927 | North | UP-1 | Barh Avarodhi | Madhukar x Sona | RV/DT | Grains: slender, white, resistant to neck blast, brown spot and blight; Yield: 25-30 Qxha. |
| 274 | IC443931 | North | UP-8 | Pant Dhan 11 | VI 206xDagi | RV/DT | Semi dwarf, grains: MS, resistant to LB, moderately resistant to BB, & BPH; Yield: 35-55 Qxha. |
| 275 | IC443929 | North | UP-6 | Pant Dhan 10 | IR-32 x Mahsuri x IR 28 | RV/DT | Semi dwarf,  grains: LS, resistant to SB, WM, WBPH, moderately resistant to BLB, Sh.B, & LB; Yield: 49-65 Qxha. |
| 276 | IC443933 | North | UP | Malviya Dhan36 | Mutant of Mahsuri | RV/DT | Grains: MS, white, resistant to major diseases; Yield: 42-45 Qxha |
| 277 | IC296050 | North | UP | Pant Dhan 957 | IR 32429-122-3-1-2xIR 31851-63-2-3-1 | RV/DT |  |
| 278 | IC443823 | North | UP | Taraori Basmati | Pureline selection from local Basmati | RV/DT | Aromatic, Tall, grains: LS, scented, Yield: 21 Qxha |
| 279 | IC443835 | North | J&K-2 | K 332 | Shenei x Norin 11 | DT |  |
| 280 | IC443836 | North | J&K-3 | SKAU 5 | China-1039 x IR-580-19-2-3-3 | DT |  |
| 281 | IC443837 | North | J&K-4 | K 429 (Kohsaar) | Shinei x Jin Masari | DT |  |
| 282 | IC443839 | North | J&K-6 | SKAU 339 |  | DT |  |
| 283 | IC443840 | North | J&K-7 | SKAU 105 | Local cultivar | DT |  |
| 284 | IC443841 | North | J&K-8 | SKAU 337 |  | DT |  |
| 285 | IC443842 | North | J&K-9 | K 475 |  | DT |  |
| 286 | IC443843 | North | J&K-10 | K 334 |  | DT |  |
| 287 | IC443844 | North | J&K-11 | CH 988 | Secondary selection from China-988 | DT | Semi dwarf (100-110 cm), grains: coarse, good quality and high head recovery |
| 288 | IC443845 | North | J&K-12 | CH 1039 | A secondary selection from China-1039 | DT | Tall (115-120 cm), grains: coarse, white, high head recovery. |
| 289 | IC469298 | North | J&K-13 | K78-13 | Shenei x Ch931 | DT |  |
| 290 | IC443834 | North | J&K-1 | Chenab (SKAU-23) | K 21-9-10-1xIR2058-521-1-1-2 | RV/DT |  |
| 291 | IC443838 | North | J&K-5 | Jehlum (SKAU-27) |  | RV/DT |  |
| 292 | IC75454 | North | PU-4 | PR 106 | (IR-8 x Peta 5) x Bella Patna | DT | Grains: LS, susceptible to BPH & GM |
| 293 | IC443907 | North | PU-1 | Basmati 370 | Pure line selection from traditional basmati | DT | Extra long grains with good cooking quality and strong aroma |
| 294 | IC443911 | North | PU-10 | Super Basmati | Basmati 320 x IR661 | DT | Semi dwarf variety, exxtra long and slender extra long and slender grains with good cooking quality and strong aroma susceptible to SB, LF & WBPH |
| 295 | IC443909 | North | PU-3 | Basmati 386 | Selection from local material | RV/DT | Tall, long and slender grains, mild aroma, suseptible to SB,BPH,LF, BB & Sh.B. |
| 296 | IC443908 | North | PU-2 | Basmati 385 | TN 1 x Basmati 370 | RV/DT |  |
| 297 | IC302930 | North | PU-7 | PR 114 | TN 1xPatong 32x PR 106x IR 8 | RV/DT | Resistant to 4 of all 8 pathotypes of BLB prevalent in Punjab |
| 298 | IC302931 | North | PU-8 | PR 115 | RP 2151-173-1-8 x PR 133 | RV/DT |  |
| 299 | IC302932 | North | PU-9 | PR 116 | PR 18 x PAU 1628 x PR 106 | RV/DT | Resistant to 4 of all 8 pathotypes of BLB prevalent in Punjab |
| 300 | IC302929 | North | PU-6 | PR 113 | IR 8x RP 2151-173-1-8x IR 8 | RV/DT | Resistant to all 8 pathotypes of BLB prevalent in Punjab |
| 301 | IC443824 | North | PU | T 23 | Selection from Kala Sukhdas | DT | Tall, grains: LS, slightly scented, Yield: 30-35 Qxha. |
| 302 | IC118780 | North | HP-2 | Himdhan | R575 x TN1 | DT |  |
| 303 | IC443825 | North | HP-3 | Himalaya1 | IR-8 x Tadukan | DT | Semi dwarf, grains: LS, fine, resistant to blast, SB and RH, moderately susceptible to Glume Blotch & BS, Yield: 38 Qxh |
| 304 | IC443826 | North | HP-4 | Himalaya 2 | Sabarmati x Ratna | DT | Semi dwarf, grains: long bold, scented, resistant to blast, SB & RH, susceptible to Glume Blotch & BS, Yield: 35 Qxha. |
| 305 | IC443827 | North | HP-5 | Himalaya 741 | CR 125-42-5 x IR2061-213 | DT |  |
| 306 | IC443830 | North | HP-8 | Himalaya 2216 | IR-8 x IR-2053-521-1-1 x IR-36 | DT | Semi dwarf, grains: LS, resistant to blast, BS, no insects-pests; Yield: 30-42 Qxha |
| 307 | IC443831 | North | HP-9 | RP 2421 | IR-36 x Kalthwar | DT |  |
| 308 | IC443829 | North | HP-7 | 'Naggardhan | Ching Sai 25 | RV/DT |  |
| 309 | IC443828 | North | HP-6 | Himalaya-799 (HPU-799) | IR 28 x Shensi Var x IR 28 | RV/DT | Semi dwarf, grains: long bold, resistant to blast, BS & Sh.R; Yield: 37-40 Qxha |
| 310 | IC443832 | North | HP | Hassan Sarai | Introduction from Iranian Basmati | RV/DT |  |
| 311 | IC118800 | West | GU-1 | GR 101 | IR-8 x P203 | DT | Aromatic |
| 312 | IC118801 | West | GU-2 | GR 102 | IR-8 x Pankhari-203 | DT | Semi dwarf (105-110 cm), grains: short bold; aromatic, Yield: 35-45 Qxha. |
| 313 | IC118802 | West | GU-3 | GR 103 | GR-11 x Mahsuri | RV/DT | Dwarf (75-95 cm), grains: fine, white, resistant to blast , SB, WBPH & leaf folder; Yield: 50-60 Qxha |
| 314 | IC393872 | West | GU | GR-9 | Sathi 34-36 x CR-544-1-2 | RV | Variety suitable for rainfed upland ecosystem |
| 315 | IC118803 | West | GU | GR-5 | Selection from local material | RV | Tall (110-115 cm), grains: medium, white, tolerant to major diseases & pests; Yield: 17-25 Q xha. |
| 316 | IC118804 | West | GU | GR-6 | GR 3 x Pusa-33 | RV |  |
| 317 | IC296145 | West | GU | Dandi | PNL-2 x IET-8320 | RV | Tall variety, coarse and bold grain, resistant to BLB, BL, S.B.,WBPH, LF |
| 318 | IC296352 | West | MA-11 | Sumati | Chadan x Pak. Basmati | DT | Salt tolerant rice variety |
| 319 | IC296364 | West | MA-7 | SYE-2001 | SYE-75 x IR-52 | DT | Dwarf variety, short bold grain, moderately resistant to blast, BLB, BS, WBPH, BPH and GM |
| 320 | IC443885 | West | MA-2 | Kundalika | Ratnagiri-24 x IET-3228 | DT | Grains: short slender, resistant to blast & major pest; Yield: 35-40 Qxha. |
| 321 | IC443886 | West | MA-3 | Pawana | Pusa-33 x IR-28 | DT | Semi dwarf, grains: LS, scented,  resistant to blast, moderately resistant to leaf scald, tolerant to major pests;  Yield: 35-40 Qxha |
| 322 | IC443887 | West | MA-4 | Phule Mawal | Pawan x Indryani | DT | Moderately resistant to blight and BLB |
| 323 | IC443888 | West | MA-5 | SKL6 | Nagpur-27 x IR-8 | DT | Semi dwarf (96 cm), grains: LS, resistant to blast & blight, moderately resistant to BLB & pests, Yield: 40-50 Qxha. |
| 324 | IC443889 | West | MA-6 | SKL7 | T(N)-1 x Basmati 370 | DT | Grains : long and slender |
| 325 | IC443890 | West | MA-8 | SYE 75 (Sindewahi) | T(N)1 x WL-112 | DT | Semi dwarf (97 cm), grains: MS, moderately resistant to blast & BLB; Yield: 45-50 Qxha. |
| 326 | IC443892 | West | MA-10 | SYE-1 | SYE-ER-1 | DT | Grains : short and bold |
| 327 | IC443893 | West | MA-12 | PKV-HMT | Selection from HMT Sona | DT | Moderately resistant to Blast, BLB |
| 328 | IC443894 | West | MA-13 | Ambe Mohar 157 | Traditional variety | DT |  |
| 329 | IC443895 | West | MA-14 | PKV-Ganesh | Daya x SKL6 | DT | Resistant to blight, bacterial blight and GM |
| 330 | IC469339 | West | MA-18 | RDN-185-2 | Halavisal-17x T(N)1 | DT | Grains : short and slender |
| 331 | IC443884 | West | MA-1 | Indrayani | Amb 157 x IR-8 | RV/DT | Aromatic, Semi dwarf, grains: LS, moderately susceptible to BLB & leaf scald, resistant to blast and major pests; Yield: 40- 45 Qxha. |
| 332 | IC469336 | West | MA-15 | Karjat-2 | Phalguna x Prakash (RPW 6-17xRP 4-14) | RV/DT | Grains: LS. |
| 333 | IC469337 | West | MA-16 | Karjat-3 | IR-36 x Karjat 35-3 | RV/DT | Grains: short bold and scented. |
| 334 | IC469338 | West | MA-17 | Karjat-4 | IR 22 x Zinia 63 | RV/DT | Short slender, superfine grain |
| 335 | IC323859 | West | MA | PKV Makarand | Indrayani x SYE 3-45 x 57 | RV | Moderately resistant to BLB, blast |
| 336 | IC443912 | West | RA-1 | Mahisughandha | BK-79 x Basmati-370 | RV/DT | Strongly aromatic, Semi dwarf (87-98 cm), grains: LS, less susceptible to SB, GM, moderately susceptible to BS, Sh.R, RTV, WBPH & BLB; Yield: 45-60 Qxha. |
| 337 | IC116269**^A^** |  | CVRC-17 | Govind | IR-20 x IR-24 | DT | Dwarf (90 cm), grains: LS, AWA, white, resistant to BLB, LB & BLS, Yield: 35-50 Qxha. |
| 338 | IC248378 |  | CVRC-14 | Tulsi | Rasi x Fine Gora | DT | Dwarf (80 cm), Grains: LS; Yield: 26-35 Qxha |
| 339 | IC75510 **^A^** |  | CVRC-2 | Jaya | T(N)1 x T-141 | DT | Dwarf (82 cm), grains: long bold, AWP, white, moderately susceptible to BLB, SB, RTV, GM and resistant to blast, Yield: 50-60 Qxha. |
| 340 | IC75514 **^A^** |  | CVRC-3 | Ratna | TKM-6 x IR-8 | DT | Dwarf (85-90 cm), grains: LS, AWA, moderately resistant to blast, LH and tolerant to SB, Yield: 45-50 Qxha |
| 341 | IC75521 |  | CVRC-6 | Swarna Dhan | Vasista x Mahsuri | DT |  |
| 342 | IC75522 |  | CVRC-5 | Sasya Sree | TKM-6 x IR-8 | DT | Semi dwarf (90-95 cm), grains: LS, AWA, white, moderately resistant to BLB and RTV, resistant to blast and *Helminthosporium*, tolerant to SB, GLH and BPH |
| 343 | IC75524 |  | CVRC-9 | Vikas | TKM-6 x IR-8 | DT | Semi dwarf (95-110 cm), grains: MS, white, tolerant to *Helminthosporium* & SB, Yield: 78 Qxha. |
| 344 | IC75526 |  | CVRC-7 | Manasarovar | RP-31-49-2 x Leb Muey Nahng | DT | Semi dwarf (105-110 cm), grains: short bold, white, resistant to blast, moderately resistant to BLB, RTV, Sh.B, GLH and BPH. |
| 345 | IC77024 |  | CVRC-4 | Rasi | (N)1 x Co.29 | DT | Semi dwarf (90-95 cm), grains: MB, white, AWA, resistant to blast, moderately resistant to RTV, Yield: 56 Qxha. |
| 346 | IC78688 |  | CVRC- 1 | IR 8 | Peta x DGWG | DT | Tall high yielding variety |
| 347 | IC296212 |  | CVRC-13 | Suraksha | Sasyasree x MR-1523 | DT | Grains: long bold; Yield: 38-43 Qxha. |
| 348 | IC296243 |  | CVRC-15 | Sonasali | RP-1015- 348-85-1 x Sona x Manoharsali | DT | Semi dwarf, grains: superfine, Yield: 30-35 Qxha. |
| 349 | IC296244 |  | CVRC-34 | Aditya | M-63-83 x Cauvery | DT | Semi dwarf (90 cm), grains: long bold, resistant to bold, tolerant to BS, BLB, RTV, susceptible to GM & BPH; Yield: 33-40 Qxha. |
| 350 | IC308319 |  | CVRC-18 | Kasturi | Basmati-370 x CRR-88-17-1-5 | DT | High-yield potential, excellent milling quality, resistance to blast and tolerance to stem border |
| 351 | IC443772 |  | CVRC-10 | Amulya | Pure line selection from Cultivar Nagani | DT | Tall (165-180 cm), grains: LS, white, moderately resistant to blast, BLB, SB, resistant to Sh.B, RTV; Yield: 30-33 Qxha. |
| 352 | IC443773 |  | CVRC-11 | Dharitiri | Pankaj x Jagannath | DT | [Resistant Varieties for rice insect pests](http://www.rkmp.co.in/content/resistant-varieties-for-rice-insect-pests) |
| 353 | IC443774 |  | CVRC-12 | Salivahana | RP5-32 x Pankaj | DT | Moderately tolerant to iron toxicity |
| 354 | IC443775 |  | CVRC-19 | Nalini | Pure line selection from Culttivar Sindurmukhi | DT | Semi deep water and salt tolerant, Tall (160 -175 cm), grains: MS, white, resistant to blast, RTV, moderately resistant to Sh.B, SB & BLB; Yield: 30-33 Qxha. |
| 355 | IC443776 |  | CVRC-20 | Pusa Basmati-1 | Pusa 150 x Karnal local | DT | High-yielding variety of scented basmati rice |
| 356 | IC113132**^A^** |  | CVRC-45 | IR-64 | IR-5857-33-2-1 x IR-2061-465-1-5-5 | DT | Semi dwarf (100 cm), grains: LS, white, resistant to blast, BLB, RTV, BPH, GLH, WBPH & GM; Yield: 58 Qxha. |
| 357 | IC443784**^A^** |  | CVRC-39 | Anjali | PR-19-2 x RR-149-1129 | RV/DT | Semi-dwarf (85-100 cm), grains: short bold, white, moderately tolerant to drought, resistant to BS, moderately resistant to blast & Sh.R; Yield: 20-30 Qxha |
| 358 | IC443777 |  | CVRC-24 | Lunisree | A mutant selection from Nonasail variety (IET-10678) | RV/DT | Tall (130 cm), grains: LS, white, no incidence of major diseases & pests; Yield: 45-50 Qxha. |
| 359 | IC469280 |  | CVRC-44 | Pooja | Vijaya x T.141 | RV/DT | Grains: MS, resistant to blast. |
| 360 | IC248514 |  | CVRC-31 | CSR 27 | Nona BokaraxIR5657-33-2 | RV/DT | Resistant to rice insect pests |
| 361 | IC308168 |  | CVRC-23 | Ajaya | IET-4141 x CR-98-7216 | RV/DT | Semi dwarf (95 cm), grains: long bold, resistant to BLB, moderately resistant to blast, SB & GM; Yield: 50 Qxha. |
| 362 | IC296248 |  | CVRC-27 | Nidhi | Sona x ARC-14529 | RV/DT | Semi-dwarf (95 cm), grains: LS, white and resistant to blast, RTV, tolerant to GLH, WBPH & BPH; Yield: 40 Qxha. |
| 363 | IC306701 |  | CVRC-28 | Triguna | Swarna Dhan x RP-1579-38 | RV/DT | Grains – long slender, tolerant to BPH & GM |
| 364 | IC443781 |  | CVRC-36 | Vasumati | PR 109 x Pakistan Basmati-1 | RV/DT |  |
| 365 | IC443785 |  | CVRC-40 | Dhanrasi | B 32 Sel. 4 x *O. rufipogon* x B 127 | RV/DT | Semi dwarf; grains - short bold; tolerant to water logged, resist. to blast, neck blast mod. resist. to BLB, BS, Sh.B, Sh.R. and RTV, GM-5, LF & SB |
| 366 | IC443780 |  | CVRC-35 | Pantdhan 16 | BG 380 x BG 367-4 BG 850-1 | RV/DT | Moderately resistant to stem borer |
| 367 | IC443779 |  | CVRC-26 | Mahamaya | Asha x Kranthi | RV/DT | Drought tolerance, Resistant to GM, tolerant to WBPH, LF, sheath rot, BS |
| 368 | IC443787 |  | CVRC-42 | Narendra Dhan 97 | N22 x Ratna | RV/DT | Dwarf (75-80 cm), resistant to many diseases & moderately resistant to, BS & Sh.R; Yield: 25-30 Qxha. |
| 369 | IC443788 |  | CVRC-43 | Narendra Dhan 359 | BG-90-2-4 x 08677 | RV/DT |  |
| 370 | IC75525 |  | CVRC-8 | Savitri (Ponmani) | Pankaj x Jagannath | RV/DT | Resistant to many insect pests |
| 371 | IC297621 |  | CVRC-22 | VL Dhan-221 | IR-2053-521-1-1-1 x Ch-1039 | RV/DT | Dwarf (90-95 cm), grains: medium, resistant to leaf & neck blast, moderately resistant to leaf scald & Sh.R, tolerant to pink stem borer & LF; Yield: 25 Qxha. |
| 372 | IC297622 |  | CVRC-29 | VL Dhan 61 | Jaya x Tapoocho-Z | RV/DT | Grains: long bold, resistant to blast. |
| 373 | IC297623 |  | CVRC-32 | VL Dhan 81 | VL Dhan 81 is a derivative of a cross China 988 x HPU 741 | RV/DT | Grains: long bold, resistant to blast. |
| 374 | IC297624 |  | CVRC-33 | Vivek Dhan 62 | China 4 x BG-367-4 | RV/DT | Grains: short bold, resistant to blast, neck blast, Sh.R, and tolerant to low temperatures; Yield: 46 Qxha |
| 375 | IC443782 |  | CVRC-37 | Vivek Dhan 82 | VL Dhan-221 x UPR-82-1-7 | RV/DT | Hilly low land and irrigated varieties |

**^A^**: Autumn rice, also known as 'Aus' in West Bengal, 'Ahu' in Assam, 'Beali' in Orissa, 'Bhadai' in Bihar, 'Virippu' in Kerala and 'Kuruvai/kar/ Sornavari' in Tamil Nadu.

**^$^**^:^ ^Hybrid rice^

**^+^**AP= Andhra Pradesh, KA= Karnataka, KE=Kerala, TN=Tamil Nadu, PY= Pondicherry, AS=Assam, ME=Meghalaya, MNP=Manipur, BI=Bihar, MP=Madhya Pradesh, OR=Orissa, WB= West Bengal, UP= Uttar Pradesh, J&K= Jammu and Kashmir, PU=Punjab, HP= Himachal Pradesh, GU=Gujarat, MA= Maharashtra, RA= Rajasthan, CVRC= Central Varietal Release and Notification Committee

*RV= Released varieties, DT= DUS tested varieties

**^@^**AWA= Abdominal White Absent, BB= Bacterial Blight, BLB= Bacterial Leaf Blight, BPH= Brown Plant Hopper, BS= Brown Spot, GH= Grass Hopper, GF= Gall Fly, GM= Gall Midge, GLH= Green Leaf Hopper, GPH= Green Plant Hopper, LB= Leaf Blight, LF= Leaf Folder, LH= Leaf Hopper, LPH= Leaf Plant Hopper, LR= Leaf Roller, MB= Medium Bold, MS=Medium Slender, RH= Rice Hispa, RLH= Rice Leaf Hopper, RTV= RTV, SB= Stem Borer, Sh.B= Sheath Blight, Sh.R= Sheath Rot, WBPH= White Back Plant Hopper
