# Supplementary material for: Comparison of SSR and SNP Markers in Estimation of Genetic Diversity and Population Structure of Indian Rice Varieties
Source: PLoS One. 2013 Dec 19;8(12):e84136. doi: 10.1371/journal.pone.0084136 (PMC3868579; doi:10.1371/journal.pone.0084136)
Supplement: Table S2 — Details of rice samples showed trait based grouping with SSR markers. (DOCX) [file pone.0084136.s002.docx]

**Table S2** Details of rice samples showed trait based grouping with SSR markers

| S. No. | Local Name | State | Trait (Biotic) |  |
| --- | --- | --- | --- | --- |
| Cluster 1c |  |  |  | |
| 163 | Ranjit | Assam | b |  |
| 98 | Karuna (Ptb 54) | Kerala | b |  |
| 325 | SYE 75 | Maharashtra | b |  |
| Cluster 2d |  |  |  |  |
| 351 | Amulya | CVR | b |  |
| 116 | ADT 37 | Tamilnadu | b |  |
|  |  |  |  |  |
