# Supplementary material for: Comparison of SSR and SNP Markers in Estimation of Genetic Diversity and Population Structure of Indian Rice Varieties
Source: PLoS One. 2013 Dec 19;8(12):e84136. doi: 10.1371/journal.pone.0084136 (PMC3868579; doi:10.1371/journal.pone.0084136)
Supplement: Table S3 — a. AOMVA analysis between Indica rice population (345 varieties) and aus rice population (29 varieties) after removing hybrid rice (1 variety) sample based on SSR marker. b. F-statistics analysis between Indica rice population (345 varieties) and aus rice population (29 varieties) after removing hybrid rice (1 variety) sample based on SSR marker. (DOCX) [file pone.0084136.s003.docx]

**Table S3a.** AOMVA analysis between *Indica* rice population (345 varieties) and *aus* rice population (29 varieties) after removing hybrid rice (1 variety) sample based on SSR marker

| Summary AMOVA Table | |  |  |  |  |  |
| --- | --- | --- | --- | --- | --- | --- |
|  |  |  |  |  |  |  |
| Source | **df** | **SS** | **MS** | **Est. Var.** | **%** |  |
| Among Pops | 1 | 20.873 | 20.873 | 0.069 | 1% |  |
| Among Indiv | 372 | 5036.217 | 13.538 | 5.801 | 74% |  |
| Within Indiv | 374 | 724.500 | 1.937 | 1.937 | 25% |  |
| Total | 747 | 5781.590 |  | 7.806 | 100% |  |

**Table S3b.** F-statistics analysis between *Indica* rice population (345 varieties) and *aus* rice population (29 varieties) after removing hybrid rice (1 variety) sample based on SSR marker

| F-Statistics | Value | P(rand >= data) |
| --- | --- | --- |
| Fst | 0.009 | 0.001 |
| Fis | 0.750 | 0.001 |
| Fit | 0.752 | 0.001 |
|  |  |  |
| Fst max | 0.572 |  |
| F'st | 0.015 |  |
