# Supplementary material for: Comparison of SSR and SNP Markers in Estimation of Genetic Diversity and Population Structure of Indian Rice Varieties
Source: PLoS One. 2013 Dec 19;8(12):e84136. doi: 10.1371/journal.pone.0084136 (PMC3868579; doi:10.1371/journal.pone.0084136)
Supplement: Table S4 — a. AOMVA analysis between Indica rice population (345 varieties) and aus rice population (29 varieties) after removing hybrid rice (1 variety) sample based on SNP marker. b. F-statistics analysis between Indica rice population (345 varieties) and aus rice population (29 varieties) after removing hybrid rice (1 variety) sample based on SNP marker. (DOCX) [file pone.0084136.s004.docx]

**Table S4a.** AOMVA analysis between *Indica* rice population (345 varieties) and *aus* rice population (29 varieties) after removing hybrid rice (1 variety) sample based on SNP marker

| Summary AMOVA Table | |  |  |  |  |
| --- | --- | --- | --- | --- | --- |
|  |  |  |  |  |  |
| Source | **df** | **SS** | **MS** | **Est. Var.** | **%** |
| Among Pops | 1 | 15.768 | 15.768 | 0.016 | 0% |
| Among Indiv | 372 | 5232.936 | 14.067 | 5.700 | 68% |
| Within Indiv | 374 | 997.500 | 2.667 | 2.667 | 32% |
| Total | 747 | 6246.205 |  | 8.383 | 100% |

**Table S4b.** F-statistics analysis between *Indica* rice population (345 varieties) and *aus* rice population (29 varieties) after removing hybrid rice (1 variety) sample based on SNP marker

| F-Statistics | Value | P(rand >= data) |
| --- | --- | --- |
| Fst | 0.002 | 0.234 |
| Fis | 0.681 | 0.001 |
| Fit | 0.682 | 0.001 |
|  |  |  |
| Fst max | 0.523 |  |
| F'st | 0.004 |  |
