# Supplementary material for: Comparison of SSR and SNP Markers in Estimation of Genetic Diversity and Population Structure of Indian Rice Varieties
Source: PLoS One. 2013 Dec 19;8(12):e84136. doi: 10.1371/journal.pone.0084136 (PMC3868579; doi:10.1371/journal.pone.0084136)
Supplement: Figure S1 — Analysis of Molecular variance (AMOVA) between Indica rice population (345 varieties) and aus rice population (29 varieties) after removing hybrid rice (1 variety) based on (a) SSR data and (b) SNP data. (DOCX) [file pone.0084136.s005.docx]

**(a)**

**Figure S1**

**(b)**
